# Hybrid autoencoder with orthogonal latent space for robust population structure inference

**DOI:** 10.1038/s41598-023-28759-x

**Published:** 2023-02-14

**Authors:** Meng Yuan, Hanne Hoskens, Seppe Goovaerts, Noah Herrick, Mark D. Shriver, Susan Walsh, Peter Claes

**Affiliations:** 1 Department of Electrical Engineering, ESAT/PSI, KU Leuven, Leuven, Belgium; 2 Department of Human Genetics, KU Leuven, Leuven, Belgium; 3 Medical Imaging Research Center, University Hospitals Leuven, Leuven, Belgium; 4 Department of Biology, Indiana University Purdue University Indianapolis, Indianapolis, IN USA; 5 Department of Anthropology, Pennsylvania State University, State College, PA USA; 6 Murdoch Children’s Research Institute, Melbourne, VIC Australia

**Keywords:** Computational biology and bioinformatics, Genetics

## Abstract

Analysis of population structure and genomic ancestry remains an important topic in human genetics and bioinformatics. Commonly used methods require high-quality genotype data to ensure accurate inference. However, in practice, laboratory artifacts and outliers are often present in the data. Moreover, existing methods are typically affected by the presence of related individuals in the dataset. In this work, we propose a novel hybrid method, called SAE-IBS, which combines the strengths of traditional matrix decomposition-based (e.g., principal component analysis) and more recent neural network-based (e.g., autoencoders) solutions. Namely, it yields an orthogonal latent space enhancing dimensionality selection while learning non-linear transformations. The proposed approach achieves higher accuracy than existing methods for projecting poor quality target samples (genotyping errors and missing data) onto a reference ancestry space and generates a robust ancestry space in the presence of relatedness. We introduce a new approach and an accompanying open-source program for robust ancestry inference in the presence of missing data, genotyping errors, and relatedness. The obtained ancestry space allows for non-linear projections and exhibits orthogonality with clearly separable population groups.

## Introduction

Population structure, or the presence of systematic differences in allele frequency and linkage disequilibrium (LD) across ancestral populations, remains an important area of research in human genetics and bioinformatics. Detection thereof can be used to infer genotypic clusters, to identify admixture, and to study the history of migration and geographic isolation^1–4^. Moreover, it allows researchers to control for confounding effects due to population stratification in genome-wide association studies (GWAS), enabling accurate genetic mapping of traits^5–7^.

Principal component analysis (PCA)^8^ is a widely used method to analyze genome-wide single nucleotide polymorphism (SNP) data, for inferring population structure^9^ and for detecting potential outliers and is routinely used as a quality control step in genetic analyses^10,11^. In general, ancestry inference using PCA consists of the following steps. First, an ancestry space is built based on either a population diverse reference dataset with known ethnicities (e.g., HapMap project^12^ or 1000 Genome project (1KGP)^13^) solely or the combined dataset of the target population and the reference dataset. In the first approach, the target samples are projected onto the learned reference space using the same set of SNP markers. Second, the population label of an unseen target sample is inferred by applying classification algorithms, or genetic ancestry can be expressed as a continuous axis of variation. However, high-quality genotype data is critical to the success of PCA^10^. In the presence of missing genotypes or errors in the target dataset, PCA has shown to produce patterns of misalignment during projection^14,15^. To overcome this problem, a robust alternative was recently proposed, known as SUGIBS, which utilizes spectral (S) decomposition of an unnormalized genomic (UG) relationship matrix generalized by an Identity-by-State (IBS) similarity matrix between the samples to be projected and individuals in the reference dataset^14^. By incorporating IBS information to correct for genotype errors and missing data during matrix decomposition and data projection, SUGIBS was proven to be more robust than PCA. In the work of SUGIBS, unnormalized PCA (UPCA) was investigated as well. Normalizing genotype data by allele frequency, typically done using PCA, causes individuals within the same population to be more alike, enhancing the distinction between populations^16^. This is beneficial for tasks such as clustering. However, normalization is challenging for heterogeneous datasets and increases sensitivity to outliers. UPCA (i.e., PCA on non-normalized data), on the other hand, can reduce sensitivity to outliers^14^. Another requisite of PCA is that reference subjects are unrelated to prevent fusing signals due to family relatedness with population structure^17^. For example, related individuals may form separate clusters far away from their respective ancestry groups if they were included in construction of the PCA reference space. Therefore, a common strategy is to first identify and then remove related individuals, which again requires more sophisticated solutions when dealing with heterogeneous and highly admixed datasets (e.g., KING-robust^18^).

In addition to matrix decomposition-based methods such as PCA, there has been a strong interest in the use of machine learning to identify ancestry groups and learn informative features based on genotype data. For example, support vector machines (SVM) have been applied to infer ancestry in an American population^19^, and singular value decomposition (SVD) was used to reduce the dimensionality of genotype data prior to training a classification neural network to perform ancestry prediction^20^. Autoencoders (AE), a type of neural network architecture capable of learning lower-dimensional latent representations in an unsupervised manner^21,22^, have been combined with clustering methods such as K-Means and hierarchical clustering to infer population structure in maize inbred lines^23^. One advantage of neural network-based approaches is that these can easily be extended and changed. Specially, their design is flexible and today many architectural alternatives as well as training strategies exist as a result of the overwhelming scientific interest in neural network-based solutions and deep learning well beyond genetics and bioinformatics. E.g., variational autoencoders (VAE), an architectural extension of AE in terms of generative capability, were able to visualize complex population structures in a two-dimensional (2D) latent space, whereas a larger number of principal components (PCs) was required for the same task^24^. Denoising autoencoders (DAE) aim to achieve more robust latent representations by using noisy data as input and trying to reconstruct the original, clean data^25,26^. DAE have been used for genotype imputation and provided accurate and robust results at different levels of missing data^27^. Aside from these two examples, many more alternatives exist, and this flexibility along with the ability to potentially learn non-linear relationships within the data makes neural network-based solutions very attractive to explore.

**Figure 1:**
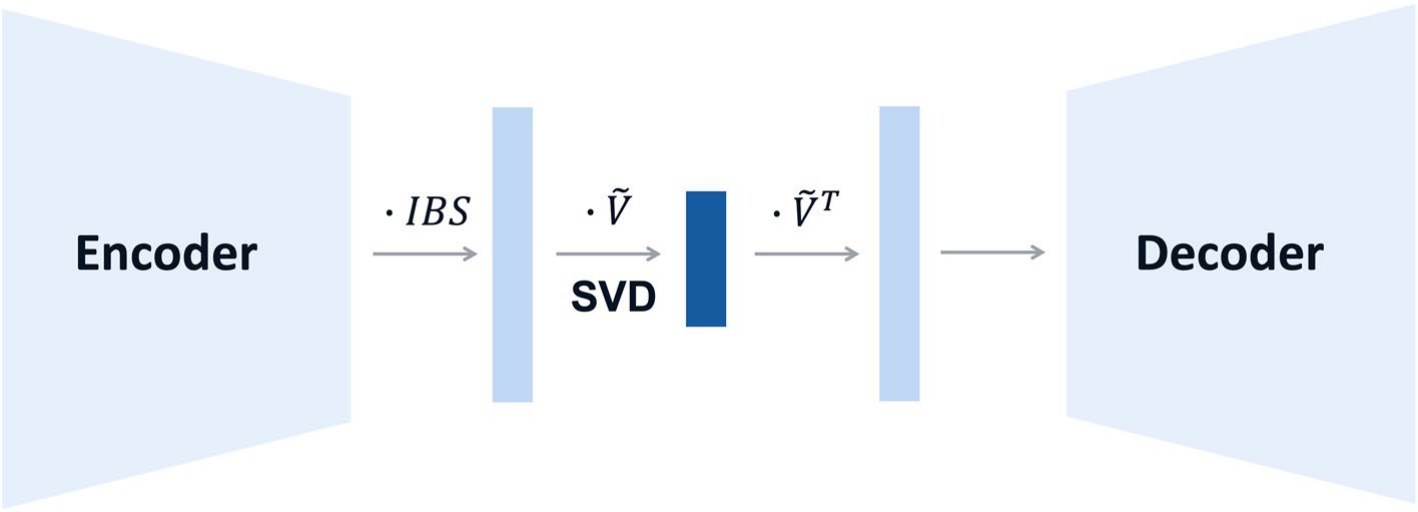
Network architecture of SAE-IBS. The encoder and decoder are fully connected neural networks. The decoder architecture is mirrored to the encoder architecture. Matrix multiplication is denoted by $\cdot$ and SVD is low-rank singular value decomposition. IBS is Identity-by-State similarity matrix.

In this work, we integrate recent advances obtained for matrix decomposition-based solutions with a neural network learning paradigm into a novel hybrid approach, referred to as SAE-IBS, which consists of a Singular Autoencoder (SAE) generalized by an IBS similarity matrix (model architecture in Fig. 1). The idea of a SAE was first investigated in a previous study on 3D shape analysis^28^. SAE was found to generate uncorrelated latent representations that code variation on independent aspects of shape variation. Moreover, it could model non-linearity in the data and offered learning-based flexibility to adapt to other applications. Inspired by this work, and the idea of merging the advantages of matrix decomposition-based methods with those from neural network-based approaches, we intend to explore the application of SAE in the context of ancestry inference. Additionally, IBS correction, as adapted from SUGIBS, is straightforwardly incorporated in this hybrid model to further boost the robustness. Our proposed model was compared with PCA and regular autoencoders through the following experiments. First, we explored the properties of the obtained low-dimensional latent representations in terms of variance–covariance structure, genetic clustering, and ancestry inference through classification. Second, we investigated the robustness of constructing an ancestry space in the presence of related individuals. Finally, we simulated missing and erroneous genotypes in the target samples and compared the robustness of different methods during the projection of target data onto a reference space. Experiments were conducted using both simulated data and real genotype data from the 1KGP^13^, the Human Genome Diversity Project (HDGP)^29^, and the Adolescent Brain Cognitive Development (ABCD) project^30^.

## Results

### Properties of the ancestry space

We explored the structure and properties of the ancestry spaces based on the 1KGP dataset for the three different and main categories of methods (PCA, AE, and SAE-IBS selected as representative of matrix-decomposition, neural-network, and hybrid models, respectively). More specifically, we visualized and investigated the obtained lower-dimensional latent space and its variance–covariance structure. Furthermore, we examined the discriminatory power of the latent representations for genetic clustering and classification.

**Figure 2:**
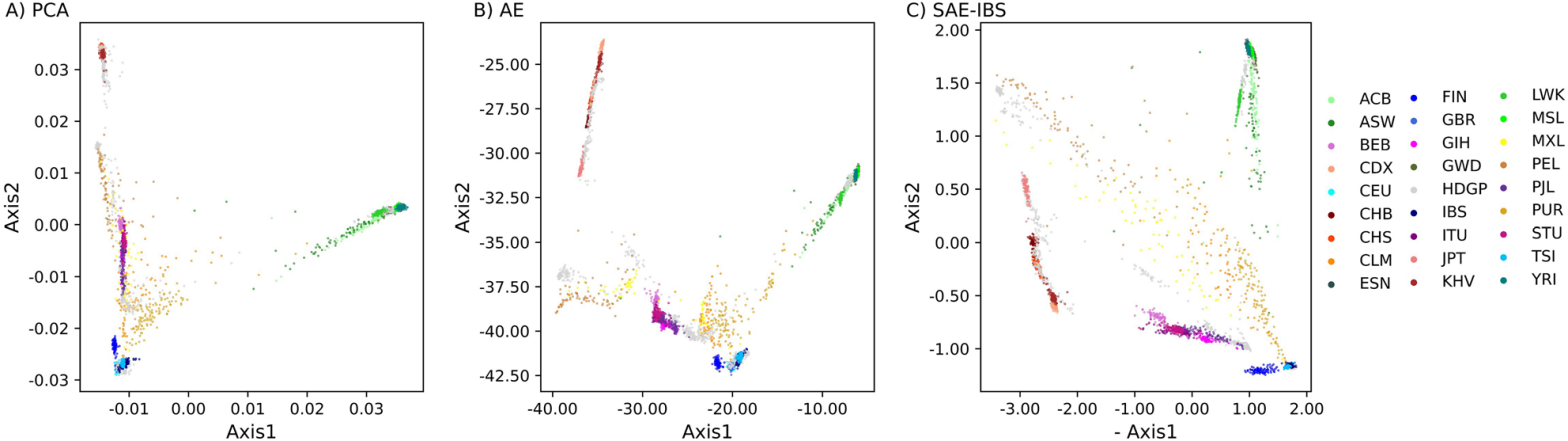
Comparison of the latent spaces for different methods. Scatter plots of the top two ancestry axes determined using (**A**) PCA, (**B**) AE, (**C**) SAE-IBS. The color of a point represents the ancestry of an individual, blue tints for European, green tints for African, red tints for East Asian, yellow tints for American, and purple tints for South Asian. The projected target samples from HDGP dataset onto 1KGP reference space are plotted in grey. Note in (**C**) SAE-IBS, to make the relative positions of clusters in different figures easier to compare visually, the first ancestry score was multiplied by − 1. African Caribbean in Barbados (ACB); African ancestry in the southwestern United States (ASW); Bengali in Bangladesh (BEB); Chinese Dai in Xishuangbanna, China (CDX); Utah residents with ancestry from northern and western Europe (CEU); Chinese in Beijing (CHB); Han Chinese South (CHS); Colombian in Medellín, Colombia (CLM); Esan in Nigeria (ESN); Finnish in Finland (FIN); British from England and Scotland (GBR); Gujarati Indians in Houston (GIH); Gambian in Western Division-Mandinka (GWD); Iberian Populations in Spain (IBS); Indian Telugu in the U.K. (ITU); Japanese in Tokyo (JPT); Kinh in Ho Chi Minh City, Vietnam (KHV); Luhya in Webuye, Kenya (LWK); Mende in Sierra Leone [MSL]; Mexican ancestry in Los Angeles (MXL); Peruvians in Lima, Peru (PEL); Punjabi in Lahore, Pakistan(PJL); Puerto Rican in Puerto Rico (PUR); Sri Lankan Tamil in the UK (STU); Nigeria; Toscani in Italy (TSI); Yoruba in Ibadan (YRI).

Figure 2 illustrates the learned ancestry spaces of PCA, AE, and SAE-IBS. The clustering pattern resembled the geographical distribution in our data sample, with Europe, East Asia and Africa situated at different points of the triangle, South Asia positioned between Europe and East Asia, while America spread out among populations because of admixture. This structure is widely observed in ancestry spaces generated by PCA, and interestingly AE and SAE-IBS also produced similar patterns. On the other hand, samples from South Asia and America overlapped in the 2-dimensional PCA space, while training AE and SAE-IBS with only two latent axes resulted in a well-structured latent space with a clear separation of the different super-populations, which was also observed in related works such as^24,31^.

#### Variance–covariance structure

**Figure 3:**
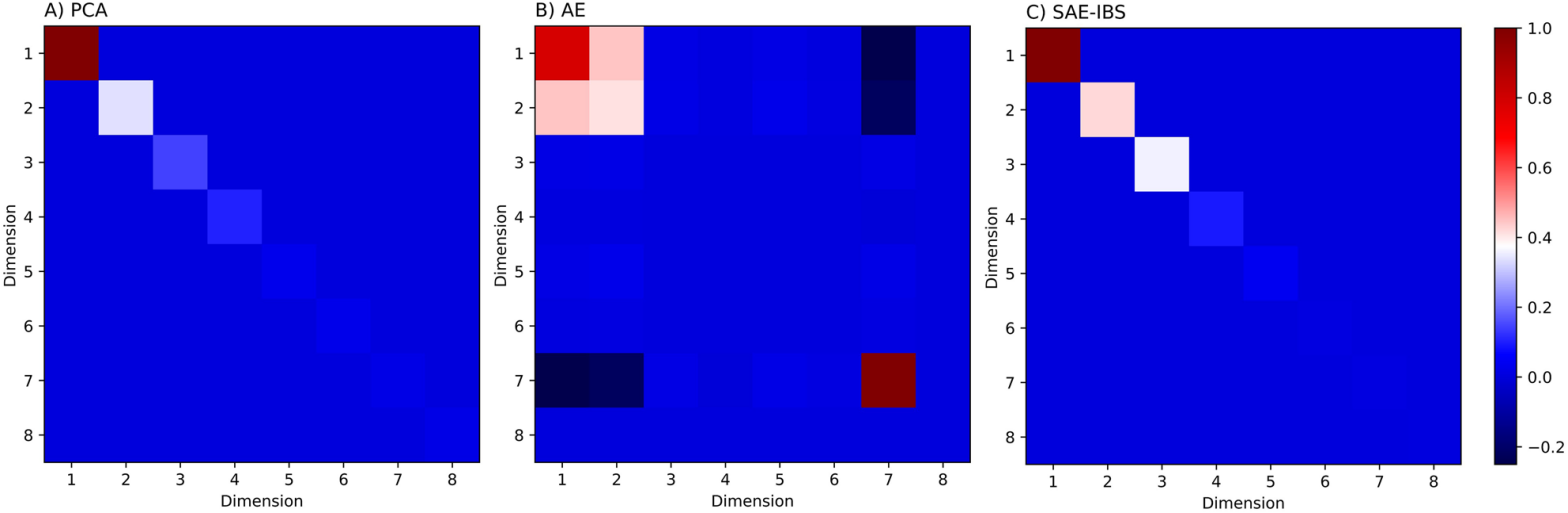
Covariance matrix of the ancestry scores for the 8-dimensional spaces constructed by (**A**) PCA, (**B**) AE, (**C**) SAE-IBS. The elements on the diagonal represent the amount of variance explained by each of the dimensions. The off-diagonal elements show the correlation between the axes along corresponding dimensions. Blue blocks indicate low (co)variance and red blocks indicate high (co)variance.

Figure 3 displays the variance–covariance matrix of the ancestry scores for 8-dimensional latent spaces constructed by PCA, AE, and SAE-IBS. For PCA and SAE-IBS, larger values appeared on the diagonal in descending order, indicating that PCA and SAE-IBS models capture most of the genotypic variation by the first few axes. The off-diagonal elements of the variance–covariance matrix represent the correlation between the different latent axes. For PCA and SAE-IBS, these were equal to zero due to the orthogonality that is enforced. In contrast, the latent axes of AE showed some degree of correlation, and the amount of variance was less concentrated in the first few dimensions.

#### Evaluation of genetic clustering performance

To evaluate the validity of the different latent representations in clustering, K-means clustering^32^ was applied to the 1KGP dataset with known population labels as ground truth information. Clustering performance was investigated at different resolutions, including super-population and sub-population scale. For this, the number of clusters in the K-means algorithm was set to 5 (the number of super-populations) and 26 (the number of sub-populations) respectively. Performance was evaluated using clustering accuracy (cluster labels compared to the known population labels). To investigate the influence of the number of ancestry axes on clustering accuracy, we built the models with varying latent dimensions: 2, 4, 8, and 12.

**Figure 4:**
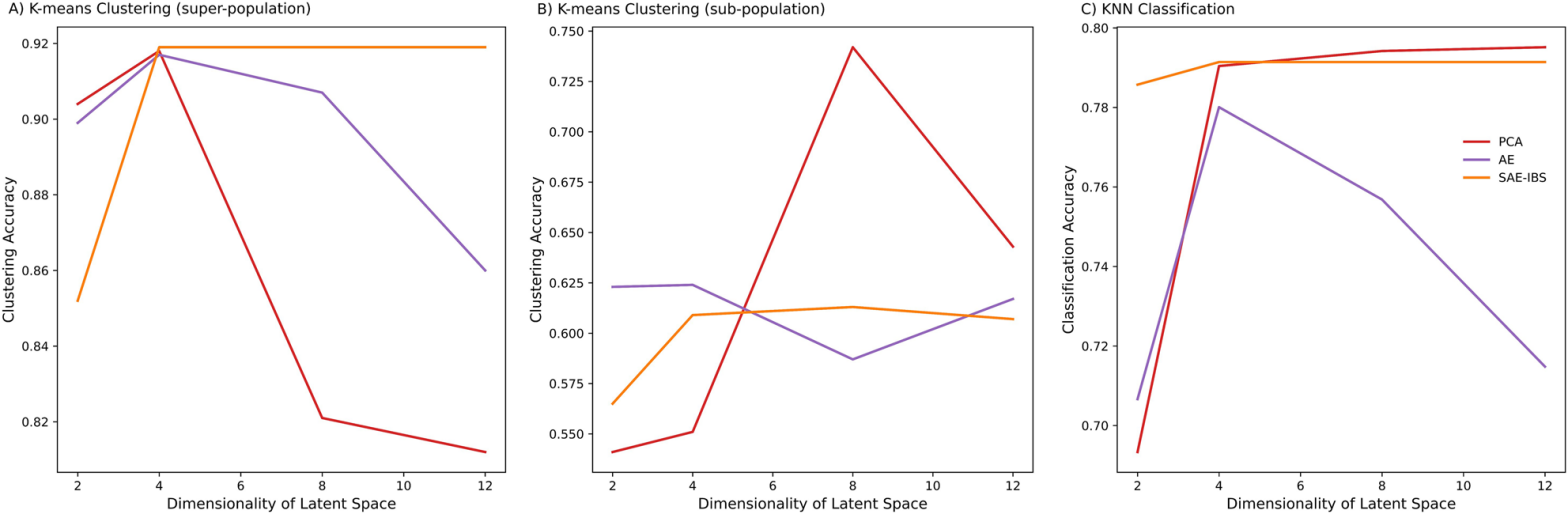
Comparison of clustering and classification accuracy under different latent space dimensions of different models. Number of clusters in K-means algorithm was set to 5 (**A**) and 26 (**B**), corresponding to the number of super-populations and sub-populations defined in the 1KGP dataset, respectively. (**C**) Number of neighbors in KNN classification was set to 3.

Figure 4A,B provide the results of clustering accuracy under different latent space dimensions of the different models. At the super-population level with latent space dimension of 2, PCA performed best, followed by the AE and SAE-IBS models. The performance of all methods increased when the latent space dimension increased from 2 to 4. However, a further increase in the number of latent dimensions reduced the performance of AE and PCA, while the performance of SAE-IBS models remained stable under different settings. The best performance of all methods was comparable and realized with 4 ancestry axes. In general, the performance for all three methods at the level of sub-population is much lower than that of the super-population. With latent space dimensions of 2 and 4, AE performed best, followed by the SAE-IBS model and PCA. The performance of PCA improved substantially when the number of latent dimensions lifted to 8, while the performance of AE and SAE-IBS models with higher latent space dimensions remained unchanged. The best performance among all approaches was realized using PCA with 8 ancestry axes. Interestingly, for PCA, the best clustering accuracy was reached with a latent space dimension set to 4 and 8 for super-population and sub-population level, respectively. This was consistent with visual inspection of the plots of subsequent PCs (Fig. S1A), in which the top four PCs captured the major continental population structure, while the subsequent PCs (PC5–PC8) accounted for additional structure in sub-populations. The latter ancestry axes of AE and SAE-IBS did not show further differentiation at the level of sub-population (Fig. S1B and C).

**Figure 5:**
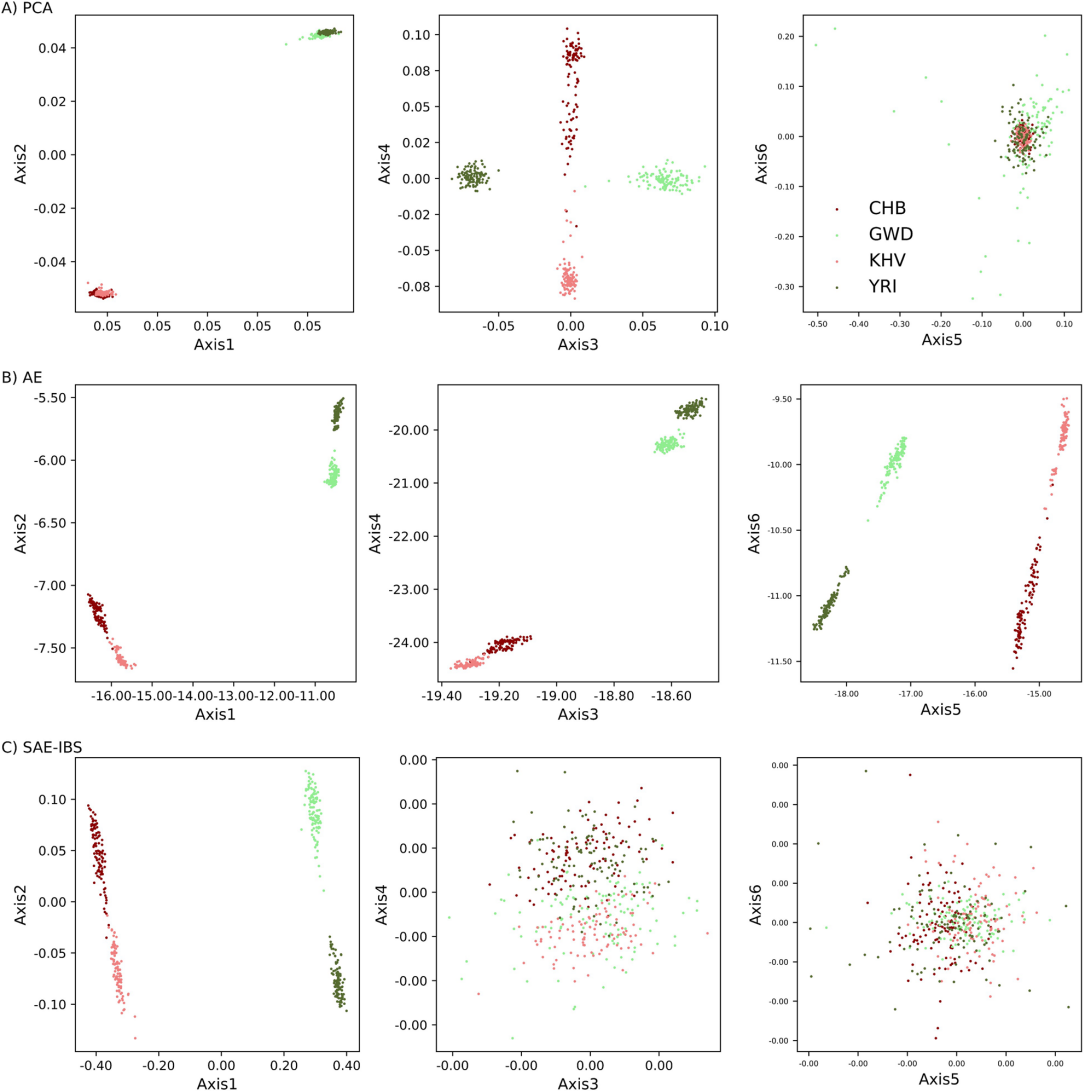
Comparison of population inference at super-population and sub-population level. The first six ancestry components for (**A**) PCA, (**B**) AE and (**C**) SAE-IBS using four sub-populations from the 1KGP dataset. The color of a point represents the ancestry of an individual, dark red for Han Chinese in Beijing, China (CHB), light red for Kinh in Ho Chi Minh City, Vietnam (KHV), light green for Gambian in Western Division, The Gambia (GWD), and dark green for Yoruba in Ibadan, Nigeria (YRI).

Although AE and SAE-IBS did not perform as optimally as PCA for clustering a large number of closely related sub-populations, this disadvantage could be overcome by analyzing sub-populations on a smaller scale. Figure 5 displays ancestry axes for experiments using individuals from four selected 1KGP sub-populations (CHB and KHV from East Asia; GWD and YRI from Africa). The first two PCA axes defined the clusters at the super-population level while the third and fourth axes further separated the sub-populations, clearly recapitulating the results from the previous experiment. In contrast, both super-population and sub-population structures were already captured by the first two latent axes of AE, further evidenced by the eigenvalues of the correlation matrix of the latent vectors of AE. Specifically, the number of eigenvalues greater than 1 (Kaiser’s rule^33^) was equal to 2, suggesting that two latent axes were adequate for clustering these four sub-populations. For SAE-IBS, the first two ancestry axes were also sufficient to reveal the hierarchical population structure, and the latter axes appeared unstructured and indistinguishable from random noise, similar to the last two axes in PCA. Furthermore, in a supplementary experiment inferencing sub-populations within one super-population (in supplementary Fig. S2), the first two axes of PCA, AE and SAE-IBS separated sub-populations similarly, and the last few axes of PCA and SAE-IBS appeared to capture noise. The latter axes of AE exhibited repeated structure from the first two axes, as evidenced by the variance–covariance, similar to the observations in Fig. 3.

#### Evaluation of ancestry inference performance

The ancestry inference performance was evaluated through the classification accuracy based on the projected ancestry scores of target data. First, we built the reference ancestry space based on the 1KGP dataset. Next, a subset of the HDGP dataset was projected onto the learned space. As shown in Fig. 2, the projected HDGP samples overlay well on the reference space. To further quantify the quality of these projections, we inferred the super-population labels for HDGP samples based on the labels of the 1KGP dataset using a K-nearest neighbors (KNN) classification^34,35^. Performance was assessed using classification accuracy. Since the exact match of sub-population labels between the two datasets is uncertain, we limited this experiment at the level of super-population. A summary table for sub-populations of the HDGP dataset used in this experiment can be found in Table S8.

Figure 4C provides the results of classification accuracy under different latent space dimensions for the different models. With the latent space dimension of 2, SAE-IBS performed best, followed by the AE and PCA models. The performance of all methods increased when the latent space dimension increased from 2 to 4. When the number of latent dimensions further increased, the performance of SAE-IBS remained stable, and the performance of PCA slightly improved, while the performance of AE reduced. The best performance of SAE-IBS and PCA was comparable and better than that of AE, obtained with 4 and 8 ancestry axes, respectively.

In addition, based on the clustering and classification experiments across different dimensions of the ancestry space, we also investigated the stability of the population structure detected. By design, PCA and related matrix-decomposition solutions each time generate the same axes, independent of the dimensionality requested, with each axis being orthogonal to the others. Specifically, the first four axes, when only 4 dimensions are sought for, are the same as the first four axes when up to 12 dimensions are sought for (Fig. S1A, Table S9). In contrast, the first four axes generated by an AE model trained with 4 and 12 dimensions respectively will differ (Fig. S1B, Table S9). PCA is a deterministic algorithm, while AE like many neural network methods is a non-deterministic algorithm. Interestingly, for SAE-IBS trained with 4 or 12 dimensions, the first four dimensions remain stable and are highly correlated (Fig. S1C, Table S9). This highlights a similar behavior of SAE-IBS compared with PCA, with orthogonality allowing ease of dimensionality selection for subsequent tasks.

### Construction of robust ancestry space in the presence of relatedness

We applied the different population structure inference methods to a subset of the heterogeneous ABCD dataset consisting of unrelated individuals (n = 5024) and 2nd degree relatives detected with a relatedness threshold of 0.0884 (two different families, one of four related individuals, and one of five related individuals). With the added flexibility in training neural network solutions (AE and SAE-IBS), we investigated training an ancestry space based on the mean squared error (MSE) loss function or L2 norm (as used in the previous experiments). This makes its sensitivity to outliers equivalent to the least-square matrix decomposition encountered in PCA. In addition, we also investigated training an ancestry space based on the mean absolute error (MAE) or L1 norm. MAE represents the mean absolute distance between the original and predicted values, while the MSE measures the average of the squared difference between the original and predicted values. The squaring means the size of the error grows quadratically as the data points are located further away from the group mean. This implies that, theoretically, MSE is more sensitive to outliers than MAE.

**Figure 6:**
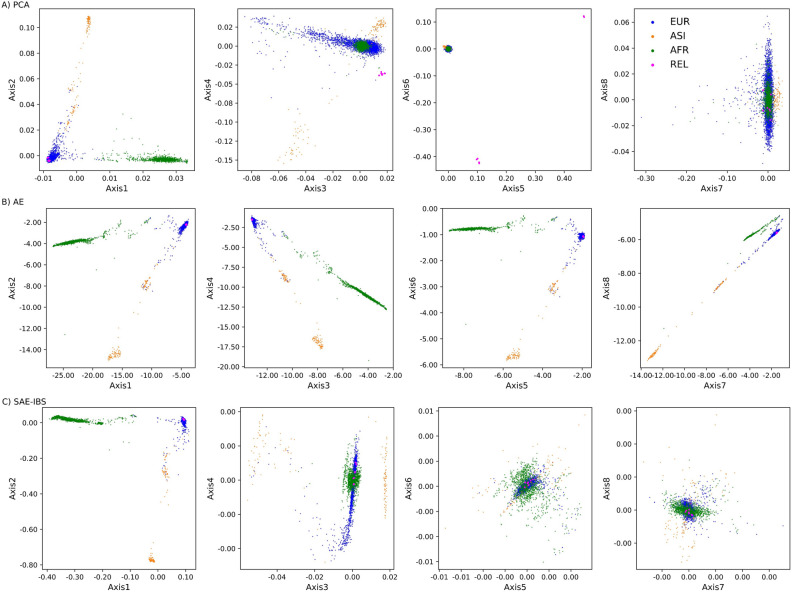
Comparison of population structure inference in the presence of related individuals. Scatter plots of the 8-dimensional ancestry space determined using (**A**) PCA, (**B**) AE, and (**C**) SAE-IBS, trained with MAE loss. The colors represent the self-reported ancestry of an individual, green for African (AFR), orange for Asian (ASI), and blue for European (EUR). Related individuals (REL) are plotted in pink.

Figure 6 displays the population structure as inferred by PCA, AE, and SAE-IBS (trained with MAE loss) with eight ancestry axes. The fifth and sixth axes of variation from the PCA model were confounded by relatedness, as shown by the related individuals that formed distinct groups far away from the main clusters. In contrast, no confounding due to relatedness was observed for AE and SAE-IBS models with MAE loss. Moreover, in a further examination with a higher latent dimension, from the 16-dimensional ancestry spaces built using AE and SAE-IBS models (Fig. S3), we did not observe any separating cluster formed by the relatives. On the other hand, the obtained ancestry axes from the AE and SAE-IBS models using MSE loss were found sensitive to relatedness (Fig. S4).

To further quantify these results, we calculated the mean Mahalanobis distance (MMD)^36^ between the related individuals and the three main population clusters on the first eight ancestry axes. Lower MMD values (Table 1) between the relatives and their matching ancestry group were found for AE and SAE-IBS models (trained with MAE loss) compared to PCA, indicating that neural network and hybrid solutions can be robust against related individuals within the dataset when trained with an MAE loss. Meanwhile, the robustness would be lost when the AE and SAE-IBS models were optimized in terms of MSE loss (Table S10).

**Table 1:** The mean Mahalanobis distance (MMD) using eight ancestry axes between the groups of relatives and three population clusters, i.e., European (EUR), African (AFR), and Asian (ASI).

Model	MMD-EUR	MMD-AFR	MMD-ASI
PCA	28.0230	151.6899	156.0633
AE	1.2370	35.7748	23.7082
SAE-IBS	1.8338	33.5951	30.9566

The relatives are of European descent.

### Robust projection of target data onto reference ancestry space

To assess the accuracy and robustness against genotype missingness and erroneousness in the target data, we constructed a reference ancestry space based on the 1KGP dataset and projected unaltered and altered HDGP samples (i.e., target data of which ancestry needs to be inferred) with simulated missing and erroneous genotypes onto the reference space. Again, with the added flexibility of neural networks, in this session we also investigated denoising mechanisms for autoencoders (DAE)^25,26^ since these are relevant when working with erroneousness input data. The hybrid models (SAE-IBS, D-SAE-IBS, D-SAE-IBS-L) were compared against matrix-decomposition based (PCA, UPCA, and SUGIBS) and neural network-based (AE, DAE, and DAE-L) alternatives. DAE-L is a proposed modification of the loss function (L) in a DAE to better enforce noise robust latent representations by adding a projection loss to the reconstruction loss. D-SAE-IBS is the denoising version of SAE-IBS, and D-SAE-IBS-L is the denoising version with an extended projection loss.

**Figure 7:**
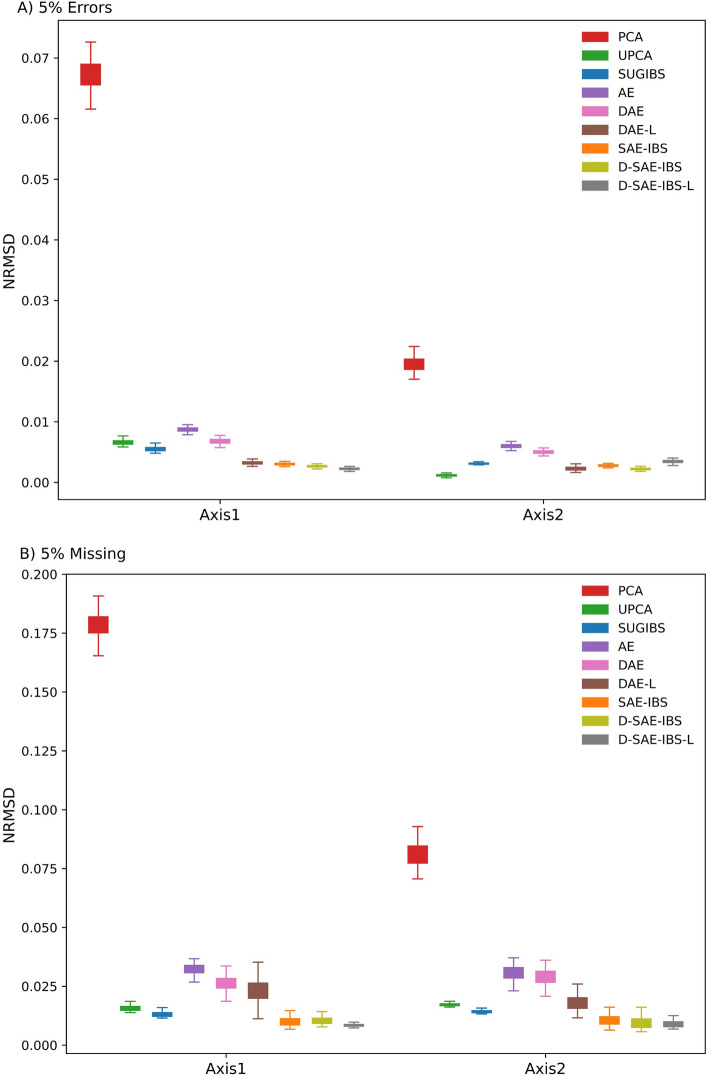
Normalized root-mean-square deviation (NRMSD) of the first two ancestry axes of different methods. (**A**) simulated erroneousness experiments and (**B**) simulated missingness experiments using matrix-decomposition methods (PCA, UPCA, SUGIBS), neural network-based methods (AE, DAE, DAE-L) and hybrid methods (SAE-IBS, D-SAE-IBS, D-SAE-IBS-L).

Figure 7 shows the normalized root mean squared deviation (NRMSD) between the ancestry scores, computed from the original (unaltered) and simulated (altered) HGDP data, on the first two ancestry axes generated by matrix decomposition-based methods (PCA, UPCA, SUGIBS), neural network-based approaches (AE, DAE, DAE-L) and hybrid models (SAE-IBS, D-SAE-IBS, D-SAE-IBS-L). The corresponding mean and standard deviation of the NRMSD scores over 100 simulations for each experiment can be found in supplementary Tables S11, S12. For the simulated erroneousness experiments, AE performed better than PCA and comparably to UPCA and SUGIBS. The denoising effect of DAE further improved the performance of AE. Moreover, DAE’s extension with an additional projection loss (DAE-L) enhanced the robustness even more. The proposed hybrid model and its extensions achieved the best performance among the above-described methods. In the scenario of simulated missingness, the denoising variants of AE (DAE and DAE-L) exhibited a similar trend in performance: by incorporating more loss terms in the objective function, the robustness increased. However, in contrast to simulated erroneousness, the group of neural network-based methods performed worse than UPCA and SUGBIS in the presence of simulated missingness. The best performance was again realized using the proposed hybrid models. Two-sample t-tests with Bonferroni correction showed significant differences in performance among most methods (p < 0.0014, Tables S13, S14). For the hybrid models, however, the gain from the denoising mechanism was not substantial. Specifically, SAE-IBS compared to using a denoising learning strategy in D-SAE-IBS, and further including an additional project loss in D-SAE-IBS-L did not significantly improve the results. Since the learning challenge is easier in SAE-IBS from a computational perspective, SAE-IBS may be a preferred method for this comparison.

Furthermore, we compared the structure of the ancestry spaces in the experiments of ancestry inference and robust projection, on account of different hyperparameter settings used. As PCA generates a lower-dimensional space via matrix decomposition, one and the same ancestry space is obtained for different tasks. In contrast, the flexibility of neural network-based solutions does require a wider exploration of network implementations, known as hyperparameter tuning in machine learning. The networks can be tunned according to research interests such as optimizing the ability to robustly project new data onto the ancestry space and optimizing the clustering/classification of the data within disparate population groups coherent with the given population labels. Implementation details can be found in supplementary (additional file 1 and Tables S1–S7). The latent space of AE customized for robust projection (Fig. S5A) was more contractive compared to that of ancestry inference experiments (Fig. 2B). For different tasks, the latent spaces of SAE-IBS (Figs. S5B and 2C) displayed a triangular shape, similar to what is typically observed for PCA in the context of population structure inference^37–39^. Besides, we found that the latent spaces of SAE-IBS under different hyperparameter settings were similar (characterized by a triangular shape and distinguishable clusters using the top two ancestry axes), while the AE latent spaces were more random and displayed great variability under different training conditions.

## Discussion and conclusion

In this work, we proposed a hybrid method for population structure inference, SAE-IBS, which yields an orthogonal latent space, like matrix decomposition-based methods, and can extract more comprehensive latent features by exploiting the non-linear nature of neural network-based methods. SAE-IBS encodes genotyping data onto a lower-dimensional space and learns to reconstruct the input data from the obtained latent representations. At the same time, it inherits important robustness properties of SUGIBS by applying SVD on the IBS generalized latent representations.

The obtained ancestry space of SAE-IBS exhibits interesting patterns: it has a PCA-like variance–covariance structure; similar to AE, different population groups are separable within fewer dimensions compared to PCA. Also, this layout remained consistent under different hyperparameter settings and was independent of the dimensionality used to train the model. This leads to a strong and desired stability of the population structure inference and an enhanced dimensionality selection after training. One can train SAE-IBS with a higher dimensionality and reduce it a posteriori without the need to retrain the network. This indicates that the fine-tuning process of SAE-IBS remains simple, and hence results are less influenced by the choice of hyperparameters and/or dimensionality. On the other hand, the latent spaces of AE and its extensions varied under different combinations of hyperparameters, meaning that additional efforts are needed for fine-tuning the AE towards ancestry inference or robustness in noisy data projections. A stronger weight decay regularization effect, i.e., more contractive latent space, is beneficial for the task of robust projection, while smaller emphasis on regularization is preferred for the purpose of ancestry inference. In addition, the generation of ancestry axes is not invariant to the dimensionality requested, and selection of dimensionality is therefore cumbersome. To make sensible choices, one often needs to compare the results using different latent dimensions.

Based on the genetic clustering and ancestry inference performance, the properties of the latent representations were further explored. PCA and SAE-IBS reached similar classification accuracy and were better than AE. Regarding the resolution of genetic clustering, AE and SAE-IBS were able to differentiate the continental clusters within a smaller number of ancestry axes compared to PCA. An advantage of PCA that we observed, is that its first few components can explain the variation at the super-population level, and the subsequent components account for the variability at the sub-population level. In contrast, AE and SAE-IBS cannot detect population structure in such a hierarchical manner and are only capable of inferring sub-population structure on a smaller selection. This could be attributed to minimizing the reconstruction error defined as the element-wise mean differences between the input and reconstructed data, which might not be a precise method to describe similarity for genotyping data. Thus, the detailed information explaining the variability within clusters is smoothed out. This argument is supported by a related work^31^, in which the LD patterns of the reconstructed and true genotype data were compared, and the AE-based method was found to experience difficulty in capturing the rare SNP variations. To achieve better clustering accuracy at the sub-population level, other types of networks specifically designed for clustering can be investigated. For instance, deep embedded clustering (DEC)^40^, which simultaneously learns feature representations and performs K-means clustering in the feature space, might be of interest for future research.

Moreover, in the experiment of constructing an ancestry space in the presence of relatedness, we show that more robust results can be obtained by simply changing the loss function from MSE to MAE. On the contrary, optimization in PCA works in a least-squares manner and hence the relatives tend to have a larger effect and form distinct clusters themselves. Robustness against relatedness is beneficial in that it could relieve some of the burden from preprocessing.

In addition to regular AE architectures, we also investigated denoising AE (DAE) architectures and proposed an extension by imposing robust projections through the incorporation of an additional loss (DAE-L). We found that these extensions could improve the robustness of AE against genotyping errors and missing data. Furthermore, these extensions were also applied to our proposed SAE-IBS model. Results showed that denoising extensions did not significantly improve the SAE-IBS model in terms of robustness against data artifacts. A possible explanation is that SAE-IBS already performs well by combining IBS information so that the gain from the denoising effect is marginal.

Nevertheless, with SAE-IBS and other neural network-based models, we have the flexibility of e.g., modifying the model architecture, adapting the loss function or using alternative training strategies. A related work^31^ applied a convolutional autoencoder with residual connections, which offers an alternative model architecture different from the fully connected autoencoder used in this work. The effect of training scheme was demonstrated for ancestry inference versus robust projection of samples. This flexibility also allows these methods to be easily adapted towards other tasks such as genotype imputation (e.g., by learning to reconstruct genotypes accurately from the input with missing values via denoising mechanisms as described in a related work^27^). Still, there is room for improvement in terms of robustness. It may be of interest for future research to explore other types of neural networks, such as denoising adversarial autoencoders^41^. These types of AE utilize an additional adversarial loss to minimize error of misclassification between actual and generated samples, which may further boost its performance.

Despite the current popularity and many advantages of neural network-based methods, hyperparameter tuning remains challenging. It is important to identify which hyperparameters to focus on in order to refine the search space. For AE models, weight decay regularization not only prevents overfitting, but also improves the robustness of projection by encouraging the feature extraction process to be locally invariant of the change of the input data. It is observed that a higher weight decay regularization results in a more robust projection. Similar ideas have been presented in the work of contractive autoencoders (CAE)^42^, in which another form of penalty term for localized space contraction was proposed. CAE has been proven to outperform AE and ensure that the parameters of the encoder are less sensitive to small variations in the input. In the case of linear activations, the loss functions of CAE and weight decay regularized AE are identical and enforcing the weights of networks to be small is the only way to have a contraction^42^. We used the Leaky ReLU activation function, which comprises two linear functions and strictly speaking is not linear. Even so, weight decay regularization in our case resembles the underlying mechanism of CAE.

While SAE-IBS exhibits desirable properties such as the orthogonal latent space, it also comes with some limitations. First, it uses a non-conventional training scheme. Namely, to speed up the learning of SAE-IBS and to provide a well-initialized embedding from the encoder to apply SVD on, we first trained an AE up to 1000 epochs and continued training SAE-IBS afterwards. Moreover, even though SAE-IBS was trained in mini batches, the SVD layer was updated based on the whole data. Specifically, within an epoch, the embeddings of each batch data from the encoder were updated and then the SVD layer was updated using the embeddings of the complete dataset. In the case of applying SVD on very large datasets, this procedure may not be feasible. This limitation motivates future work to replace SVD by incremental SVD^43^, which efficiently updates the SVD outcome when new data becomes available. Second, in theory non-linear relationships within the data can be captured by using non-linear neural networks instead of linear transformers. However, this does not guarantee that all non-linearities are mapped out from the data, as input to the linear SVD layer. In other words, more work along the lines of explainable AI is needed to better understand the transition from non-linear to linear layers in the model. Lastly, but most importantly, the application of SAE-IBS is limited to study designs where reference genotype data is available for computing the IBS similarity matrix between the target and reference data. This is not required for PCA, AE, DAE and their non-IBS extensions. As an alternative, a standard SVD can be proposed onto the latent representations, turning the eigenvalue decomposition generalized by the IBS matrix into a non-generalized and thus standard eigenvalue decomposition. This will still generate an orthogonal latent representation within an AE architecture and was first explored in related work encoding 3D palate shape variations^28^. However, robustness against genotyping errors and missing data is then not obtained.

Although this work focuses on the robustness in population inference given erroneous input data, there are certainly other applications of neural network-based and hybrid methods worthy of future investigations. Specifically, incorporating variational loss allows to better sample from the ancestry space and to generate artificial genotyping data, suggested by a related work^24^. Another type of generative networks, generative adversarial networks (GAN), have also been applied to create artificial human genomes^44^. It would thus be interesting to explore these models for genotype generation and possibly combine them with SAE models and IBS information.

In conclusion, the proposed SAE-IBS model is a new and hybrid approach that integrates the advantages of matrix-decomposition (PCA and SUGIBS) and neural network-based (AE) solutions into ancestry inference from genome-wide genotype data. By incorporating IBS information, like SUGIBS, it was shown to be more robust when projecting poor quality target samples onto a reference ancestry space. Furthermore, like AE and in contrast to PCA, our approach can construct a robust ancestry space in the presence of relatedness by training based on MAE loss. Interestingly, like PCA and in contrast to AE, the stability, and therefore repeatability, of the ancestry inference is very acceptable and due to the presence of orthogonality in the latent representation dimensionality selection can be done more readily. Moreover, the learned latent representations reflect various properties of the data such as cluster identities. Our method performs equally well as the existing methods for genetic clustering and ancestry inference at the super-population level. Like AE, different population groups are separable within fewer dimensions compared to PCA. This is beneficial in terms of data compression and visualization. Finally, the proposed method inherits the flexibility of neural network-based methods, which allows it to be easily adapted for other applications and multi-task learning.

## Material and methods

### Genotyping and data processing

The datasets used in this study are (1) the 1KGP^13^ dataset, consisting of 2504 individuals from 26 populations in Europe (EUR), Africa (AFR), East Asia (EAS), South Asia (SAS), and the Americas (AMR); (2) the HDGP^29^ dataset with 1043 individuals from 51 worldwide populations; and (3) a subset from the ABCD^30^ dataset, consisting of 5033 individuals of European, African, and Asian descent.

We followed the preprocessing steps described in^14^. In brief, for the HGDP dataset, we first converted the assembly from the NCBI36 (hg18) to the GRCh37 (hg19) using the NCBI Genome Remapping Service. For the 1KGP dataset, related individuals were detected using the KING-robust kinship estimator^18^ with a relatedness threshold of 0.0442, corresponding to the 3rd degree relatives, after which one individual in each group of relatives was randomly retained. For the 1KGP and HGDP datasets, individuals with more than 10% missing genotypes were removed. Pruning based on LD of SNPs is recommended for PCA^9^. We performed multiple rounds of LD pruning with a window size of 50, a moving step of 5, and ${r}^{2}$ cutoff of 0.2 until no additional SNPs were removed. We then intersected the 1KGP and HGDP datasets to extract the set of overlapping SNPs and match their alternate alleles, resulting in a final set of 155,243 SNPs for analyses.

For the ABCD dataset, the same procedures for removing missing genotypes and LD pruning were conducted, resulting in a final set of 137,482 SNPs for analyses. Individuals with missing self-reported ancestry were removed. Three ancestry groups were selected, i.e., Asia (ASI), Europe (EUR), and Africa (AFR). The related individuals in the ABCD dataset were detected using the KING-robust kinship estimator^18^ with a relatedness threshold of 0.0884, corresponding to the 2nd degree relatives. In total, 3564 individuals were found to have at least one relative within the dataset. There were 1692 pairs, 57 trios, one family comprising 4 siblings, and one family comprising 5 siblings. We retained the related individuals that had more than three relatives. The final set consisted of 5024 unrelated individuals and 9 related individuals (European descent).

### Hybrid model

The general architecture of our proposed approach is displayed in Fig. 1. SAE-IBS, like AE, consists of an encoder network and a decoder network, and makes use of SVD and IBS information correcting for potential data artifacts, like SUGIBS. Detailed descriptions of the models used for comparison can be found in supplementary (additional file 1).

Let $X\in {\mathbb{R}}^{{n}_{1}\times m}$ denote the unnormalized genotype matrix with additive genotype coding (aa =  − 1, Aa = 0, AA = 1 and missing = − 2), and $S\in {\mathbb{R}}^{{n}_{1}\times {n}_{1}}$ denote the pairwise IBS similarity matrix of $X$, which is calculated following the rules in Table S1. The similarity degree of an individual is defined as ${d}_{ii}=\sum_{j}^{{n}_{1}}{s}_{ij}$ where ${s}_{ij}$ is the IBS similarity between individual $i$ and any other individual $j$ in the reference dataset. The similarity degree matrix is a diagonal matrix defined as $D=diag\left\{{d}_{11},\dots , {d}_{{n}_{1}{n}_{1}}\}\right.$. During training, the outputs from the last layer of the encoder are multiplied by the IBS similarity degree matrix ${G= D}^{-\frac{1}{2}}f(WX +b)$ where $G\in {\mathbb{R}}^{{n}_{1}\times h}$ is IBS generalized features, $f(\cdot )$ is a non-linear function with the weight matrix $W$ and the bias vector $b$. Then, the fully connected layers linking the encoder and decoder with the bottleneck, which are typically used in regular AE, are now replaced by a low-rank SVD to ensure orthogonal latent representations. This yields $G= \widetilde{U}\widetilde{\Sigma }{\widetilde{V}}^{T}$ in which $\widetilde{\Sigma }\in {\mathbb{R}}^{k\times k}$ is a square diagonal matrix with its diagonal entries being the non-zero singular values of $G$ and the semi-orthogonal matrices $\widetilde{U}\in {\mathbb{R}}^{{n}_{1}\times k}$ and $\widetilde{V}\in {\mathbb{R}}^{h\times k}$ contain the left and right singular vectors, respectively. The latent representation is given by $\widetilde{Z}=\widetilde{U}\widetilde{\Sigma }$, and the corresponding loading matrix is denoted by $\widetilde{V}.$ Next, the decoder maps the latent representation back to a reconstruction output $\widehat{X} = g(W^{\prime}\widetilde{Z}{\widetilde{V}}^{T} +b^{\prime})$ where $g(\cdot )$ is a non-linear function with the weight matrix $W^{\prime}$ and the bias vector $b^{\prime}$. The network is then trained to minimize the reconstruction error. The objective function takes the form

$${J}_{SAE-IBS}= \sum_{x}L\left(x, g\left({ D}^{-\frac{1}{2}}f(x)\widetilde{V}{\widetilde{V}}^{T}\right)\right)$$

Given an unseen dataset with ${n}_{2}$ individuals and the same set of SNPs as the reference dataset, let $Y\in {\mathbb{R}}^{{n}_{2}\times m}$ denote its unnormalized genotype matrix. The reference similarity degree is defined as ${\widetilde{d}}_{ii}= \sum_{j}^{{n}_{2}}{\widetilde{s}}_{ij}$ where ${\widetilde{s}}_{ij}$ is the IBS similarity between the $i$ th individual in the unseen dataset and the $j$ th individual in the reference dataset. The reference IBS similarity degree matrix is defined as $\widetilde{D}=diag\left\{{\widetilde{d}}_{11},\dots , {\widetilde{d}}_{{n}_{2}{n}_{2}}\}\right.$. Then, the projected scores of the unseen data onto the reference space can be obtained by ${\widetilde{D}}^{-1}f(WY +b)\widetilde{V}$.

Like AE, many different variants of the general SAE-IBS architecture exist. We can extend the SAE-IBS model to a denoising version as first proposed in DAE^25,26^, referred to as D-SAE-IBS, by using simulated and perturbated noisy inputs and learning to reconstruct the original noise-free data input. Despite a DAE aims to denoise input, i.e., enforcing noiseless output, it does not guarantee learning noise-robust latent features. It is very possible that a clean and noisy input data sample are projected onto different latent representations, but still generate the same “noiseless” output. From this motivation, we propose to include an extra robust projection loss (mathematical details in additional file 1) in the original objective function, yielding a denoising singular autoencoder generalized by Identity-by-State matrix with modified loss, referred to as D-SAE-IBS-L.

### Implementations

#### Training strategy

Neural network-based methods are often criticized for their danger of overfitting, i.e., the model fits extremely well to training data but fails to generalize to unseen data. To overcome this problem, we trained the models with weight-decay regularization by default. Furthermore, we applied early stopping^45^ to monitor the training process and determine the optimal number of epochs. Before training, the input dataset was divided into training and validation sets (90% of samples for training, 10% for validation). After each epoch of training, we evaluated the model performance on the validation set. Training stops at the point when no improvement is observed on the validation set over a given number of epochs. The number of epochs to wait for is referred to as early stopping patience. This ensures that the training is not terminated too soon, considering the stochastic nature of training a neural network. Details of regularization, early stopping, and the customized training strategy for the hybrid models are described in supplementary (additional file 1).

To accelerate the training process, the additive coded genotype data were first normalized so that the values are bounded within the interval of [0,1]. The encoder and decoder networks are fully connected networks. We defined the reconstruction error as the MSE between the original genotypes and their reconstruction by default, and MAE loss was used in the experiment involving related individuals. We experimented with different model architectures, i.e., varying the number of hidden layers and the number of hidden units per layer. The model hyperparameter controlling the emphasis on projection loss $\beta$ was also fine-tuned. The empirical analyses and detailed model architectures are described in supplementary (additional file 1).

#### Genetic clustering

We used the K-means clustering function from the scikit-learn package. The ‘n_init’ parameter, defining the number of times to run the algorithm with different centroid seeds, was set to 1000. All other parameters were kept by default (init = 'kmeans + +’, max_iter = 300, tol = 0.0001, verbose = 0, copy_x = True, algorithm = 'auto').

Following^40^, the evaluation metric of clustering performance, i.e., clustering accuracy is defined by $accuracy= \underset{\mathit{perm}\in P}{\mathrm{max}}\frac{\sum_{i=1}^{q}1\left\{{c}_{i}=perm(\widehat{{c}_{i}})\right\}}{q}$ where ${c}_{i}$ and $\widehat{{c}_{i}}$ are the given true label and the predicted class label, respectively. The best assignment of the predicted labels can be found via permutation. $P$ is the set of all possible permutations (in total, K! permutations) in [1, K], where K is the number of clusters. The Hungarian algorithm^46^ helped to compute the best mapping between true and predicted labels efficiently, in $O({K}^{3})$.

#### Classification of target populations

We used the K-nearest neighbors classification function from the scikit-learn package. The number of neighbors was set to 3. All other parameters were kept by default (weights = 'uniform', algorithm = 'auto', leaf_size = 30, p = 2, metric = 'minkowski'). Classification accuracy is defined by $accuracy = \frac{\sum_{i=1}^{q}1\left\{{c}_{i}=\widehat{{c}_{i}}\right\}}{q}$ where ${c}_{i}$ and $\widehat{{c}_{i}}$ are the given true label and the predicted class label, respectively.

#### Related individuals

In general, the top ancestry axes inferred by PCA can capture the population structure, and the latter axes might be confounded by relatedness. Therefore, the number of ancestry axes was set to 8. To quantify the distance in the multivariate ancestry space between a sample and the main population cluster, we computed the Mahalanobis distance using all 8 ancestry axes. Then, we took the mean Mahalanobis distance of all relatives as the evaluation metric of population inference accuracy.

#### Experiments of simulated artifacts

As shown in^14^, the difference of the top eight ancestry axes across different methods exhibit similar patterns. Therefore, the number of axes to be inferred in our experiments was set to 2 for simplicity reasons only. We followed the experimental strategy described in^14^, which simulates 5% genotype missingness and 5% genotyping errors (e.g., from aa to Aa or AA) of the rare SNPs (minor allele frequency < 0.05). We applied the operation of genotype masking (missingness) and alternating (errors) on the HDGP dataset to generate 100 datasets for the experiments of missingness and erroneousness, respectively. Then, the datasets with simulated noise were projected onto the 1KGP reference space. We computed the root mean square deviations (RMSD) between the top ancestry scores generated using the original (unaltered) and simulated (altered) datasets. Furthermore, to ensure comparability across methods, we normalized RMSD by the range of the scores using the original dataset, resulting in NRMSD.

To train denoising autoencoders, we generated 100 partially corrupted 1 KG datasets in terms of genotype masking and alternating. In D-SEA-IBS and D-SEA-IBS-L, the IBS similarity degree matrix is calculated between the partially corrupted 1 KG datasets and the target dataset. To formally test the significance of difference between different methods, we applied two-sample t-tests with Bonferroni correction on the NRMSD of the different methods over the 100 simulations. There are in total 36 independent tests, leading to a Bonferroni adjusted p-value of 0.0014 (i.e., 0.05/36).

### Ethics statement

The datasets used in this study are openly and publicly accessible, with broad consent for research purposes.

## Supplementary Information

Supplementary Information.

## Data Availability

The following publicly available datasets were used in this study. 1000 Genome Project dataset https://www.internationalgenome.org/. Human Genome Diversity Project dataset: https://www.cephb.fr/hgdp/. Adolescent Brain Cognitive Development dataset: https://nda.nih.gov/abcd. The implementation of SAE-IBS and other neural network-based methods were written in Python and available at https://github.com/mm-yuan/SAE-IBS. PCA, UPCA, SUGIBS, and simulations of erroneousness and missingness were performed using SNPLIB, a MATLAB toolbox, which can be found at https://github.com/jiarui-li/SNPLIB. The transition between human genome reference assembly was conducted via NCBI Genome Remapping Service: https://www.ncbi.nlm.nih.gov/genome/tools/remap.
