# Supplementary material for: Hybrid autoencoder with orthogonal latent space for robust population structure inference
Source: Sci Rep. 2023 Feb 14;13:2612. doi: 10.1038/s41598-023-28759-x (PMC9929087; doi:10.1038/s41598-023-28759-x)
Supplement: Supplementary file 1 — Supplementary Information. [file 41598_2023_28759_MOESM1_ESM.pdf]

Supplementary

Additional file 1 — Methods and Implementations

Population Structure Inference Methods

Matrix Decomposition-Based Methods

Principal Component Analysis

In the context of genetic data, PCA aims to explain the variation in allele frequencies by finding a low-dimensional linear transformation that maximizes the projected variance. To solve the PCA problem, we performed SVD on the normalized genotype matrix. Given n_1 individuals and m SNPs, let $X \in \mathbb{R}^{n_1 \times m}$ denote the unnormalized genotype matrix with additive genotype coding (aa=−1, Aa=0, AA=1 and missing=-2). The normalized genotype is obtained by $x_{ir}^{norm} = \frac{x_{ir} - 2a_r}{\sqrt{2a_r(1-a_r)}}$, where x_{ir} and x_{ir}^{norm} are the unnormalized genotype and normalized genotype at SNP r for individual i, respectively, and a_r is the sample allele frequency for SNP r. Then SVD takes as input the normalized genotype matrix $X_{norm} \in \mathbb{R}^{n_1 \times m}$ and decomposes it into a product of three matrices $X_{norm} = U\Sigma V^T$ where $\Sigma \in \mathbb{R}^{n_1 \times m}$ is a diagonal matrix of size $m \times n_1$ containing the singular values and the orthogonal matrices $U \in \mathbb{R}^{n_1 \times n_1}$ and $V \in \mathbb{R}^{m \times m}$ contain the left and right singular vectors, respectively. The dimension of the input data is then reduced by projecting it onto a space spanned by the top k singular vectors. Let $U_k \in \mathbb{R}^{n_1 \times k}$ and $\Sigma_k \in \mathbb{R}^{k \times k}$ denote the left singular vectors and the singular values of the first k principal components, then the input data in its lower dimensional representation is given by $U_k\Sigma_k$, and the corresponding loading matrix is denoted by $V_k \in \mathbb{R}^{m \times k}$. The projected scores of unseen data can be obtained by multiplication of the normalized genotype matrix with V_k.

Unnormalized Principal Component Analysis

UPCA works similarly to PCA, except that SVD takes the unnormalized genotype matrix as input. Interpopulation variation is captured from the second PC onwards, while the first PC represents an average SNP pattern, as is common for PCA on non-centered data. Therefore, the first PC in UPCA can be omitted.

Spectral Decomposition Generalized by Identity-by-State Matrix

SUGIBS was previously proposed as a robust alternative against laboratory artifacts and outliers[14] by applying SVD on the IBS generalized genotype matrix, where IBS information corrects for potential artifacts due to errors and missingness.

Let $S \in \mathbb{R}^{n_1 \times n_1}$ denotes the pairwise IBS similarity matrix of the unnormalized genotype matrix X, which is calculated following the rules in Table S1. The similarity degree of an individual is defined as $d_{ii} = \sum_j^{n_1} s_{ij}$ where s_{ij} is the IBS similarity between individual i and

any other individual j in the reference dataset. The similarity degree matrix is a diagonal matrix defined as $D = diag\{d_{11}, \dots, d_{n_1 n_1}\}$. SUGIBS works similarly to PCA, except that the IBS generalized genotype matrix $D^{-\frac{1}{2}}X$ is used as input for performing SVD, i.e., $D^{-\frac{1}{2}}X = U\Sigma V^T$. Likewise, to UPCA, the first component of SUGIBS aggregates the average SNP pattern and can therefore be omitted. For the projection of unseen samples, we use the second component and onwards $V_k' = \{v_2, \dots, v_{k+1}\}$ where v_k is the kth right singular vector.

Given an unseen dataset with n_2 individuals and the same set of SNPs as the reference dataset, let $Y \in \mathbb{R}^{n_2 \times m}$ denote its unnormalized genotype matrix. The reference similarity degree is defined as $\tilde{d}_{ii} = \sum_j^{n_2} \tilde{s}_{ij}$ where $\tilde{s}_{ij}$ is the IBS similarity between the ith individual in the unseen dataset and the jth individual in the reference dataset. The reference similarity degree matrix is defined as $\widetilde{D} = diag\{\tilde{d}_{11}, \dots, \tilde{d}_{n_2 n_2}\}$. The unseen dataset can be projected onto the reference space following $\widetilde{D}^{-1}YV_k'$.

Neural Network-Based Methods

Autoencoder

An autoencoder consists of two parts: an encoder network and a decoder network. The encoder maps input data to a latent representation $Z = f(WX + b)$; the decoder maps the latent representation back to a reconstruction output $\hat{X} = g(W' Z + b')$ where $f(\cdot)$ and $g(\cdot)$ are nonlinear functions, W and W' are the weight matrix, b and b' are the bias vector, X, $\hat{X}$ and Z are the input data, the reconstructed data and the latent representation, respectively. The network is then trained to minimize the reconstruction error. The objective function takes the form

$$J_{AE} = \sum_x L\left(x, g\big(f(x)\big)\right)$$

where L is the reconstruction error.

Regularized Autoencoder

To reduce overfitting of the model and improve its performance, regularization-based methods are often used. One widely used regularization is weight-decay [43], which favors small weights by optimizing the following regularized objective function

$$J_{AE-wd} = \sum_x L\left(x, g\big(f(x)\big)\right) + \lambda \sum_w W^2$$

where hyperparameter λ controls the strength of the regularization. This encourages sparse weight matrix and thus reduces the redundancy.

Denoising Autoencoder

In a denoising autoencoder [25,26], the initial input is partially corrupted before training, and then sent through the network. Based on the encoding and decoding of the corrupted input

data, it is desirable to predict the original, uncorrupted data as its output. This yields the following objective function:

$$J_{DAE} = \sum_{x} \mathbb{E}_{\tilde{x}\sim q(\tilde{x}|x)} \left[L\left(x, g\big(f(\tilde{x})\big)\right)\right]$$

where the corrupted version $\tilde{x}$ of original input x is obtained through the process $q(\tilde{x}|x)$.

Denoising Autoencoder with Modified Loss

An additional term favoring robust mapping at the bottleneck/latent space is included in the original objective function of DAE, yielding the following loss function:

$$J_{DAE-L} = \sum_{x} \mathbb{E}_{\tilde{x}\sim q(\tilde{x}|x)} \left[L\left(x, g\big(f(\tilde{x})\big)\right)\right] + \beta L\big(f(x), f(\tilde{x})\big)$$

where hyperparameter β controls the emphasis on noise-free projections. The objective now is to learn latent representations that are not only robust for reconstruction, but also at the same time robust for projection.

Implementation Details

The encoder and decoder networks are fully connected feed-forward networks with Leaky ReLU [48] activation functions connecting each layer, except for the last layer of the decoder sigmoid activation is used to ensure the output values are bounded between [0 1]. We used the Adam optimizer [49] with an initial learning rate of 0.001. To allow the optimizer to take smaller steps when training gets close to convergence, we applied a learning rate scheduler to reduce the learning rate of the optimizer by 0.9999 after every epoch. To fit in available GPU memory (11,019MiB), we trained the networks in mini batches of 256 samples. The models are implemented and trained on an NVIDIA GeForce RTX 2080 Ti using PyTorch 1.7.

To implement the early stopping mechanism, we track if the validation loss keeps improving. If the difference of the validation loss between two epochs is below 0.1, it is quantified as no improvement. The early stopping patience was set to be 300 epochs and the maximum number of epochs equaled 3000 when training AE and SAE-IBS. For denoising extensions, every 25 epochs we generated a different simulated noisy dataset and fed to the model, therefore we relaxed the max epoch (to 5000) when training DAE, DAE-L, D-SAE-IBS, and D-SAE-IBS-L. To speed up the learning of SAE-IBS (and its denoising extensions) and to provide a well-initialized embedding from the encoder to apply SVD on, we pre-trained an AE firstly with up to 1000 epochs and continued training SAE-IBS afterward.

Following the suggestions by [50], we experimented with several parameter configurations in two steps: the first one involves the number of layers and the number of hidden units; the second one investigates emphasis on projection loss β. If not explicitly stated otherwise, recommended values by default in PyTorch 1.7 [51] were used for any other hyperparameters (amsgrad: False, betas: [0.9, 0.999], eps: 1e-08).

Firstly, the final hyperparameter configuration of the AE model with latent space dimension of 2 was decided. As shown in Table S2, the configuration in bold was selected as the final setting for the experiments of robust projection because it resulted in the smallest validation loss and NRMSD for the simulated missingness experiments, and relatively small NRMSD for the simulated erroneousness experiments. The same procedure was conducted for other tasks and their final settings are listed in Table S3. Then, to ensure fair comparison, the same settings were used when training AE with higher latent space dimensions, denoising variants of AE, and hybrid models. Furthermore, for the experiments of robust projection using DAE-L, we fine-tuned the hyperparameter defining the emphasis on projection loss β based on NRMSD (Table S4 and Table S5). Similarly, this parameter was tuned for D-SAEIBS-L and the final settings are displayed in Table S6 and Table S7.

Table S1. Identity-by-state similarity

IBS	AA	Aa	aa
**AA**	2	1	0
**Aa**	1	2	1
**aa**	0	1	2
**N/A**	0	0	0

Table S2. Comparison of different model architectures using AE with latent space dimension of 2 and weight decay of 0.01 for the experiments of robust projection. *The hyperparameter configuration in bold was selected as the final setting.*

Model	Architecture	Validation loss	NRMSD missing	NRMSD error
AE	2-layer {128, 128}	4673.00	0.0640	0.0079
AE	2-layer {512, 128}	4668.56	0.0692	0.0091
AE	3-layer {64, 64, 64}	4705.96	0.0432	0.0068
**AE**	**3-layer {128, 128, 128}**	**4665.65**	**0.0315**	**0.0074**
AE	3-layer {512, 128, 64}	4669.04	0.0424	0.0053
AE	4-layer {128, 128, 128, 128}	4660.43	0.0355	0.0070

Table S3. The Final hyperparameter configurations for different tasks.

Experiment	Architecture	Weight decay
Robust projection	3-layer {128, 128, 128}	1e-2
Clustering	3-layer {512, 128, 64}	1e-9
Inference with Relatedness	3-layer {640, 240, 80}	1e-8

Table S4. Fine-tune the hyperparameter defining the emphasis on projection loss β for the experiment of simulated erroneousness using DAE-L. *The hyperparameter configuration in bold was selected as the final setting.*

Hyperparameter β	NRMSD
10	0.0032
50	0.0046
100	0.0052
**200**	**0.0028**
500	0.0032
1000	0.0040

Table S5. Fine-tune the hyperparameter defining the emphasis on projection loss β for the experiment of simulated missingness using DAE-L. *The hyperparameter configuration in bold was selected as the final setting.*

Hyperparameter β	NRMSD
10	0.0390
**50**	**0.0207**
100	0.0276
200	0.0479
500	0.0294
1000	0.0366

Table S6. The Final configurations for the experiment of simulated erroneousness.

Model	Architecture	Weight decay	Hyperparameter β
AE	3-layer {128, 128, 128}	1e-2	-
DAE	3-layer {128, 128, 128}	1e-2	-
DAE-L	3-layer {128, 128, 128}	1e-2	200
SAE-IBS	3-layer {128, 128, 128}	1e-2	-
D-SAE-IBS	3-layer {128, 128, 128}	1e-2	-
D-SAE-IBS-L	3-layer {128, 128, 128}	1e-2	100

Table S7. The Final configurations for the experiment of simulated missingness.

Model	Architecture	Weight decay	Hyperparameter β
AE	3-layer {128, 128, 128}	1e-2	-
DAE	3-layer {128, 128, 128}	1e-2	-
DAE-L	3-layer {128, 128, 128}	1e-2	50
SAE-IBS	3-layer {128, 128, 128}	1e-2	-
D-SAE-IBS	3-layer {128, 128, 128}	1e-2	-
D-SAE-IBS-L	3-layer {128, 128, 128}	1e-2	1

Additional file 2 — Additional Figures

Figure S1. Ancestry spaces of (A) PCA, (B) AE and (C) SAE-IBS for latent space dimensions equaling 4 and 12 in the experiments of clustering and classification. *The first 4 latent axes of the SAE-IBS model trained with a latent space dimension of 12 are mostly equal to the latent axes of the SAE-IBS model trained with a latent space dimension of 4. On the other hand, there is no clear pattern of latent spaces obtained using AE. The color of a point represents the ancestry of an individual, blue tints for European, green tints for African, red tints for East Asian, yellow tints for American, and purple tints for South Asian. African Caribbean in Barbados (ACB); African ancestry in the southwestern United States (ASW); Bengali in Bangladesh (BEB); Chinese Dai in Xishuangbanna, China (CDX); Utah residents with ancestry from northern and western Europe (CEU); Chinese in Beijing (CHB); Han Chinese South (CHS); Colombian in Medellín, Colombia (CLM); Esan in Nigeria (ESN); Finnish in Finland (FIN); British from England and Scotland (GBR); Gujarati Indians in Houston (GIH); Gambian in Western Division – Mandinka (GWD); Iberian Populations in Spain (IBS); Indian Telugu in the U.K. (ITU); Japanese in Tokyo (JPT); Kinh in Ho Chi Minh City, Vietnam (KHV); Luhya in Webuye, Kenya (LWK); Mende in Sierra Leone [MSL]; Mexican ancestry in Los Angeles (MXL); Peruvians in Lima, Peru (PEL); Punjabi in Lahore, Pakistan(PJL); Puerto Rican in Puerto Rico (PUR); Sri Lankan Tamil in the UK (STU); Nigeria; Toscani in Italy (TSI); Yoruba in Ibadan (YRI).*

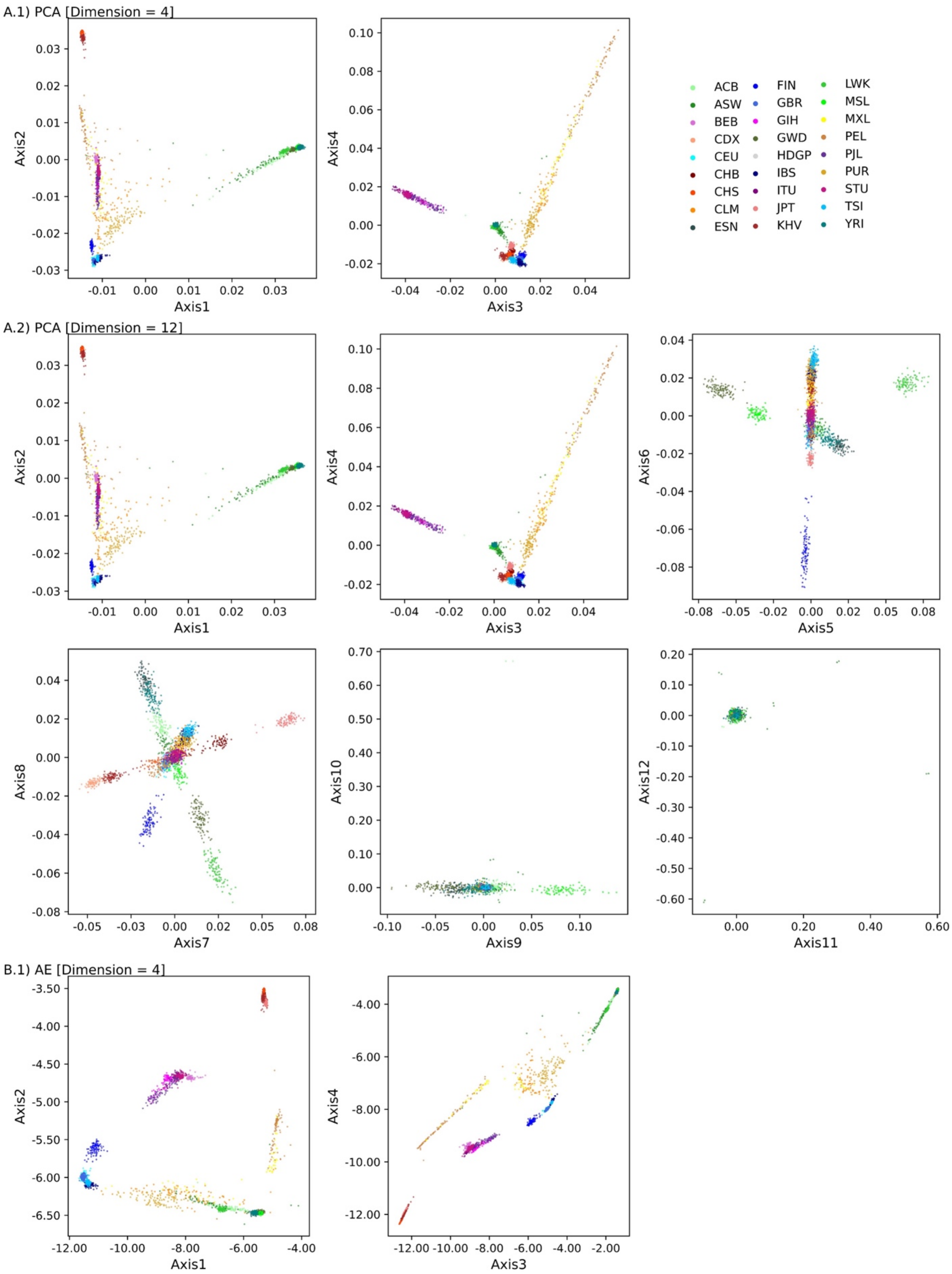
A.1) PCA [Dimension = 4]
Axis1
Axis2
Axis3
Axis4
ACB
ASW
BEB
CDX
CEU
CHB
CHS
CLM
ESN
FIN
GBR
GIH
GWD
HDGP
IBS
ITU
JPT
KHV
LWK
MSL
MXL
PEL
PJL
PUR
STU
TSI
YRI
A.2) PCA [Dimension = 12]
Axis5
Axis6
Axis7
Axis8
Axis9
Axis10
Axis11
Axis12
B.1) AE [Dimension = 4]

B.2) AE [Dimension = 12]

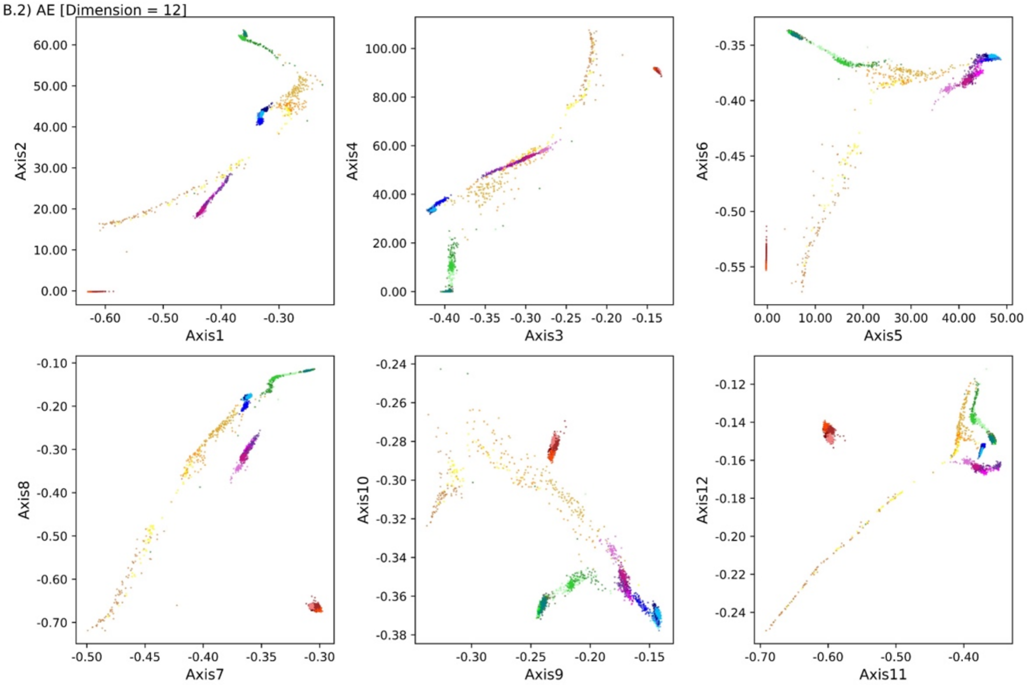

C.1) SAE-IBS [Dimension = 4]

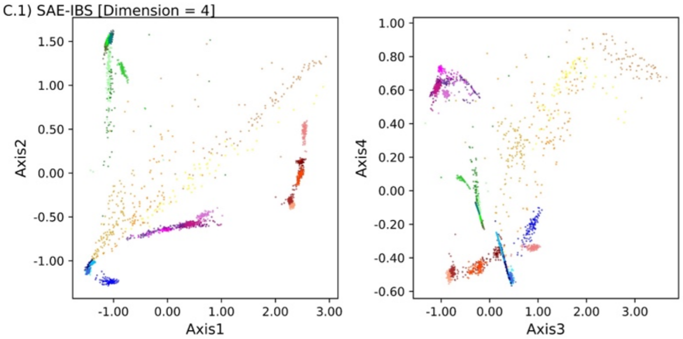

C.2) SAE-IBS [Dimension = 12]

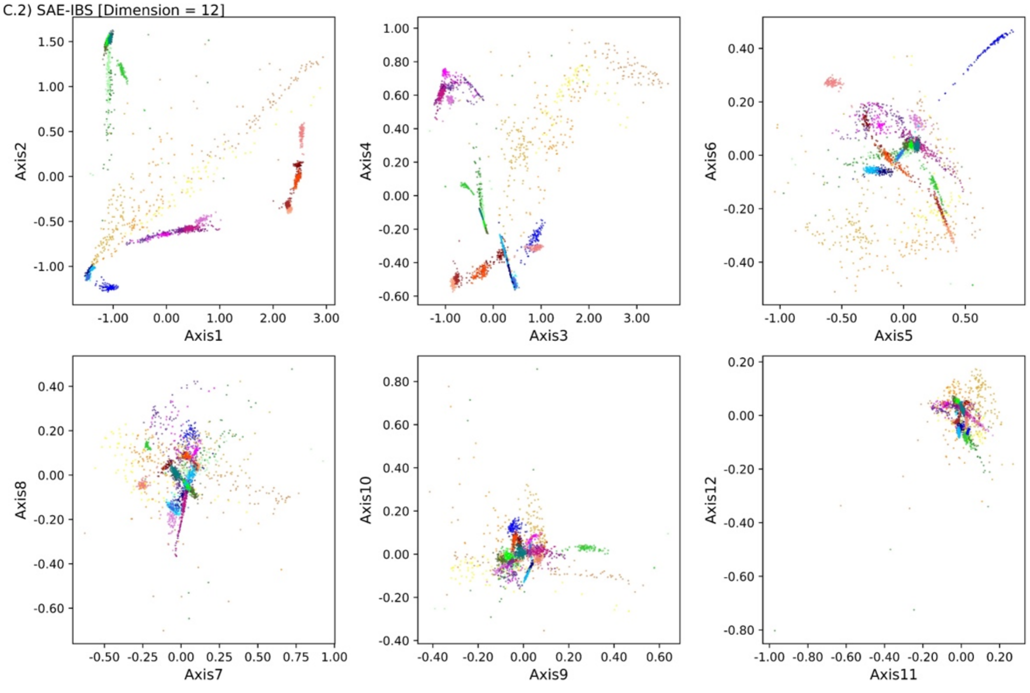

Figure S2. Ancestry spaces of (A) PCA, (B) AE and (C) SAE-IBS for the experiment inferencing sub-populations within one super-population. *The color of a point represents the ancestry of an individual, blue for Iberian Populations in Spain* (*IBS*), *green for Finnish in Finland* (*FIN*); *red for Toscani in Italy (TSI); orange for northern and western Europe (CEU), and purple for British* from *England and Scotland* (*GBR*).

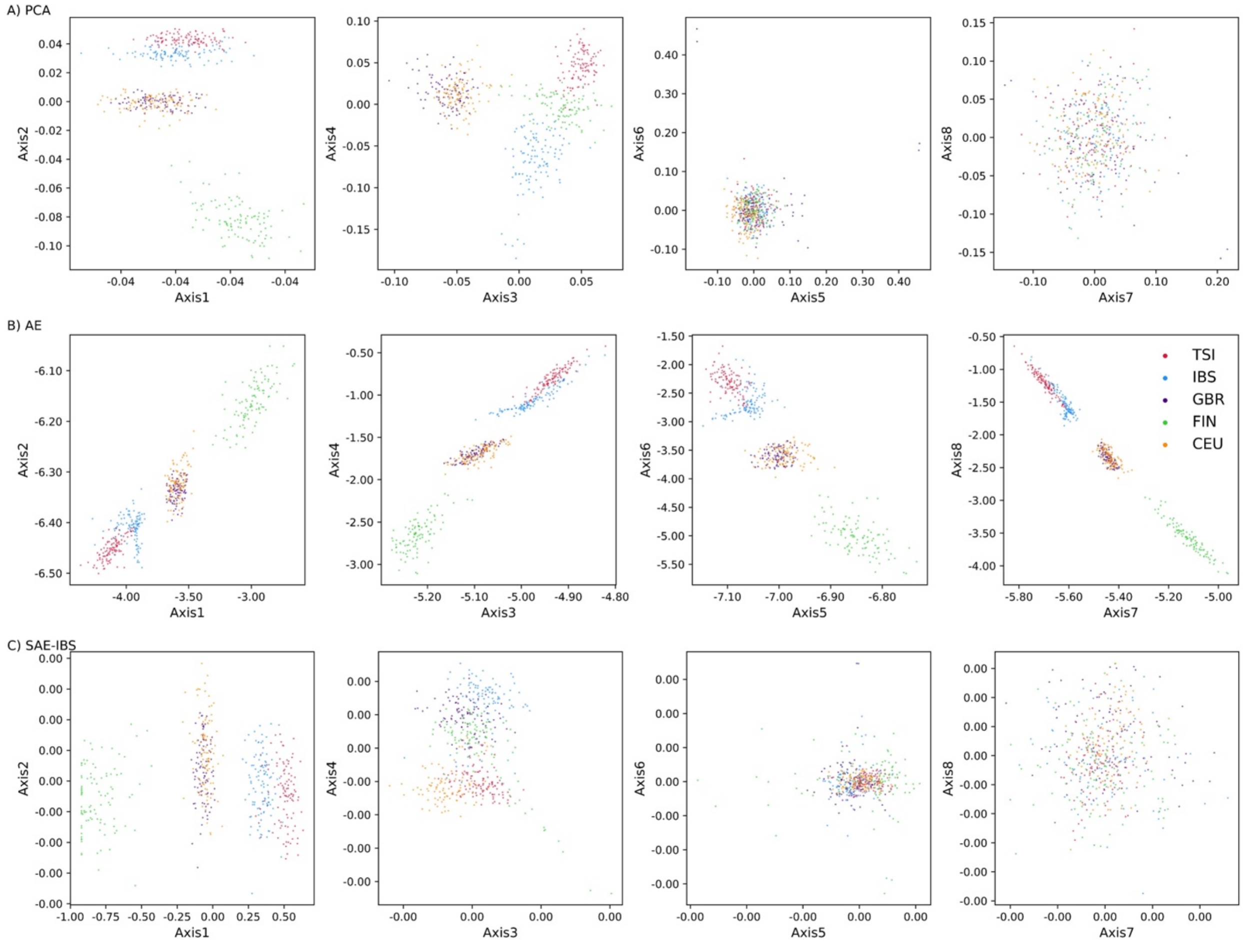

Figure S3. Comparison of population structure inference in the presence of related individuals. *Scatter plots of the 16-dimensional ancestry space determined using (A) AE and (B) SAE-IBS, trained with MAE loss. The colors represent the self-reported ancestry of an individual, green for African (AFR), orange for Asian (ASI), and blue for European (EUR). Related individuals (REL) are plotted in pink.*

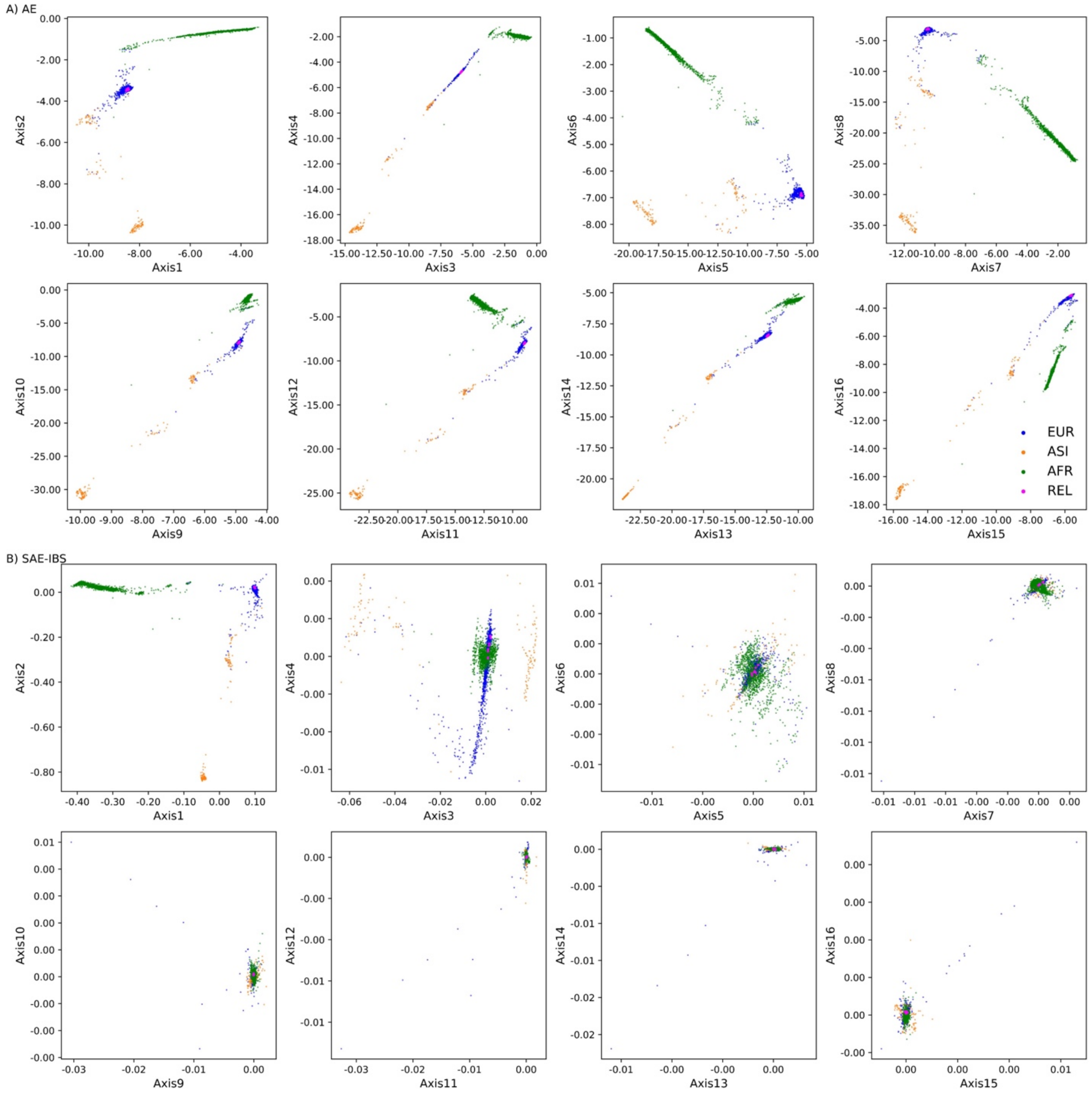

Figure S4. Comparison of population structure inference in the presence of related individuals. *Scatter plots of the 8-dimensional ancestry space determined using (A) AE and (B) SAE-IBS, trained with MSE loss. The colors represent the self-reported ancestry of an individual, green for African (AFR), orange for Asian (ASI), and blue for European (EUR). Related individuals (REL) are plotted in pink.*

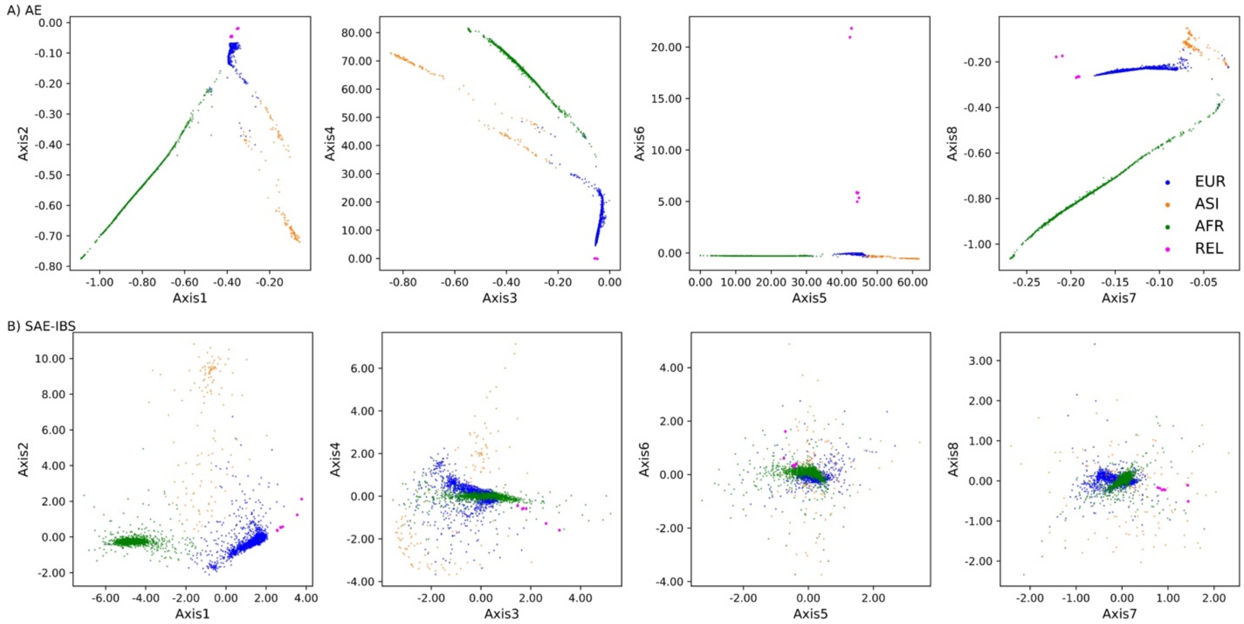

Figure S5. Ancestry spaces of (A) AE and (B) SAE-IBS in the experiment of robust projection.

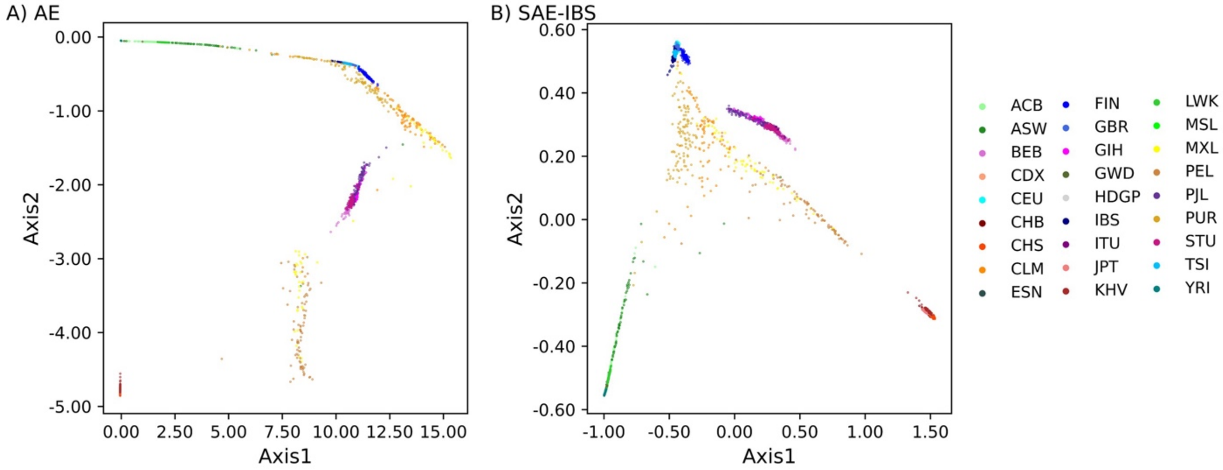

Figure S6. Comparison of clustering accuracy under different latent space dimensions of different models. *Number of clusters in K-means algorithm was set to 5 (A) and 26 (B), corresponding to the number of super-populations and sub-populations defined in the 1KGP dataset, respectively. Condition 1 and 2 corresponds to hyperparameter settings of ancestry inference and robust projection, respectively.*

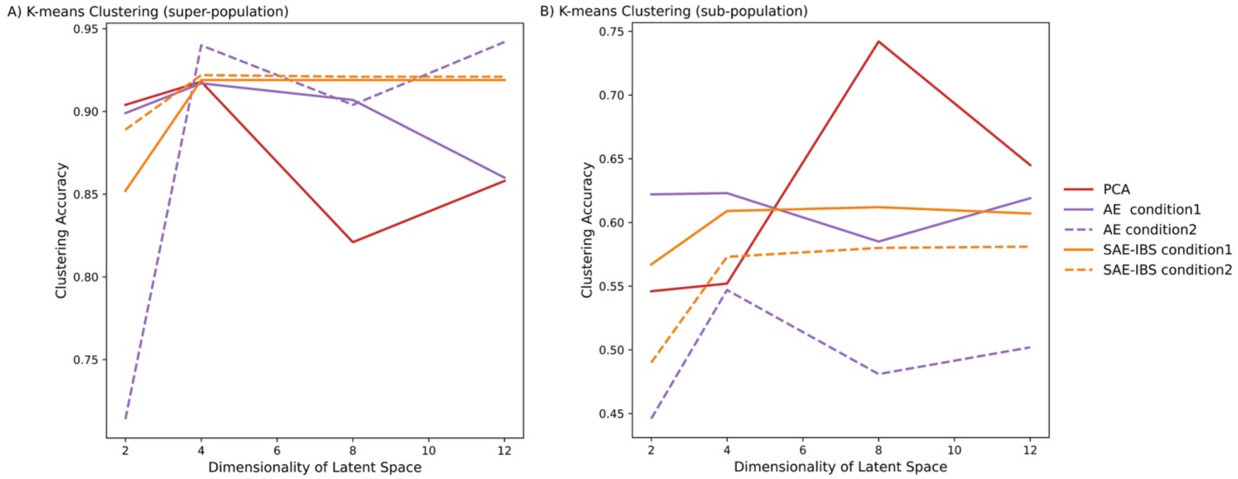

Additional file 3 — Additional Tables

Table S8. Summary table of the subset of HDGP dataset used in the experiment of population structure inference.

Region	Geographic Origin	Ethnicity	Sample Count
**Africa**	Central African Republic	Biaka Pygmy relatives	**127**
	Democratic Republic of Congo	Mbuti Pygmy relatives	
	Senegal	Mandenka relatives	
	Nigeria	Yoruba relatives	
	Namibia	San relatives	
	Kenya	Bantu NE relatives	
	S. Africa Bantu S.E.	Bantu S.E. Pedi	
	S. Africa Bantu S.E.	Bantu S.E. Sotho	
	S. Africa Bantu S.E.	Bantu S.E. Tswana	
	S. Africa Bantu S.E.	Bantu S.E. Zulu	
	S. Africa Bantu S.W.	Bantu S.W. Herero	
	S. Africa Bantu S.W.	Bantu S.W. Ovambo	
**South Asia**	Pakistan	Brahui	**210**
	Pakistan	Balochi relatives	
	Pakistan	Hazara relatives	
	Pakistan	Makrani	
	Pakistan	Sindhi relatives	
	Pakistan	Pathan	
	Pakistan	Kalash relatives	
	Pakistan	Burusho	
	China	Uygur (minority)	
**East Asia**	China	Han	**216**
	China	Tujia (minority)	
	China	Yizu (Yi) (minority)	
	China	Miaozu (Miao) (minority)	
	China	Oroqen (minority) relatives	
	China	Daur (minority)	
	China	Mongola (minority)	
	China	Hezhen (minority)	
	China	Xibo (minority)	
	China	Dai (minority)	
	China	Lahu (minority) relatives	
	China	She (minority)	
	China	Naxi (minority) relatives	

	China	Tu (minority)	
	Japan	Japanese	
	Cambodia	Cambodian relatives	
**Europe**	France	French (various regions) relatives	**119**
	France	Basque	
	Italy	Sardinian	
	Italy	from Bergamo	
	Italy	Tuscan	
	Orkney Islands	Orcadian relatives	
**America**	Mexico	Pima (relatives)	**108**
	Mexico	Maya (relatives)	
	Colombia	Piapoco and Curripaco relatives	
	Brazil	Karitiana (relatives)	
	Brazil	Surui (relatives)	

Table S9. Pearson correlation coefficient between ancestry axes obtained from models trained with different latent space dimensions (4 or 12). *For instance, the correlation between the first axes obtained from AE trained with 4 and 12 dimensions equaled to 0.2223.*

**Model**	**Pearson Correlation Coefficient**			
	**Axis1**	**Axis2**	**Axis3**	**Axis4**
**PCA**	1	1	1	1
**AE**	0.2223	0.9251	-0.8839	-0.0031
**SAEIBS**	1	1	1	0.9995

Table S10. The mean Mahalanobis distance (MMD) using eight ancestry axes between the groups of relatives and three population clusters, i.e., European (EUR), African (AFR), and Asian (ASI). *The relatives are of European descent.*

**Model**	**MMD-EUR**	**MMD-AFR**	**MMD-ASI**
**PCA**	28.0230	151.6899	156.0633
**AE(MAE)**	1.2370	35.7748	23.7082
**AE(MSE)**	21.0173	5.0988E+03	8.8426E+03
**SAE-IBS(MAE)**	1.8338	33.5951	30.9566
**SAE-IBS(MSE)**	7.5770	71.9296	30.5254

Table S11. Mean and Standard deviation of the NRMSD scores over 100 simulations for the experiment of erroneousness.

	Axis1		Axis 2	
	**Mean**	**Standard deviation**	**Mean**	**Standard deviation**
**PCA**	0.0673	0.0022	0.0195	0.0014
**UPCA**	0.0066	3.9817E-04	0.0012	2.1174E-04
**SUGIBS**	0.0055	3.6401E-04	0.0031	1.0581E-04
**AE**	0.0087	3.7404E-04	0.0060	3.2220E-04
**DAE**	0.0068	3.9920E-04	0.0050	2.4139E-04
**DAE-L**	0.0032	2.4832E-04	0.0023	2.9995E-04
**SAE-IBS**	0.0030	1.9897E-04	0.0028	1.8795E-04
**D-SAE-IBS**	0.0027	1.9118E-04	0.0022	2.0186E-04
**D-SAE-IBS-L**	0.0022	1.6171E-04	0.0035	2.2449E-04

Table S12. Mean and Standard deviation of the NRMSD scores over 100 simulations for the experiment of missingness.

	Axis1		Axis 2	
	**Mean**	**Standard deviation**	**Mean**	**Standard deviation**
**PCA**	0.1788	0.0050	0.0811	0.0050
**UPCA**	0.0158	0.0014	0.0172	6.7652E-04
**SUGIBS**	0.0133	0.0013	0.0143	7.7523E-04
**AE**	0.0322	0.0023	0.0307	0.0032
**DAE**	0.0266	0.0035	0.0290	0.0035
**DAE-L**	0.0229	0.0047	0.0184	0.0038
**SAE-IBS**	0.0101	0.0019	0.0107	0.0022
**D-SAE-IBS**	0.0104	0.0016	0.0099	0.0031
**D-SAE-IBS-L**	0.0085	8.0456E-04	0.0093	0.0021

Table S13. Results of the two-sample t-tests on the NRMSD of different methods over 100 simulations for the experiment of erroneousness. *Bonferroni correction method was applied to compute the adjusted significance level, accounting for multiple compassion. The non-significant p-values (p>0.0014) are marked in red.*

	Axis1		Axis 2	
	Mean Difference	p-value	Mean Difference	p-value
**PCA vs. UPCA**	0.0607	0	0.0183	0
**PCA vs. SUGIBS**	0.0618	0	0.0164	0
**PCA vs. AE**	0.0585	0	0.0135	0
**PCA vs. DAE**	0.0605	0	0.0145	0
**PCA vs. DAE-L**	0.0640	0	0.0172	0
**PCA vs. SAE-IBS**	0.0642	0	0.0167	0
**PCA vs. D-SAE-IBS**	0.0646	0	0.0173	0
**PCA vs. D-SAE-IBS-L**	0.0650	0	0.0160	0
**UPCA vs. SUGIBS**	0.0011	1.2611E-19	-0.0019	4.4342E-112
**UPCA vs. AE**	-0.0021	1.2661E-66	-0.0048	0
**UPCA vs. DAE**	-2.0548E-04	1	-0.0038	7.7335E-273
**UPCA vs. DAE-L**	0.0034	1.1542E-134	-0.0011	2.1016E-44
**UPCA vs. SAE-IBS**	0.0036	3.1128E-146	-0.0016	1.8354E-83
**UPCA vs. D-SAE-IBS**	0.0039	1.0473E-166	-0.0011	3.7461E-41
**UPCA vs. D-SAE-IBS-L**	0.0043	6.2900E-191	-0.0023	1.8843E-142
**SUGIBS vs. AE**	-0.0032	1.4808E-127	-0.0029	4.9692E-197
**SUGIBS vs. DAE**	-0.0013	3.6642E-27	-0.0019	4.0913E-110
**SUGIBS vs. DAE-L**	0.0023	3.7091E-73	8.2638E-04	4.4238E-26
**SUGIBS vs. SAE-IBS**	0.0025	4.5398E-84	3.3114E-04	2.5712E-04
**SUGIBS vs. D-SAE-IBS**	0.0028	4.2642E-104	8.7243E-04	7.5773E-29
**SUGIBS vs. D-SAE-IBS-L**	0.0033	1.0215E-128	-3.4381E-04	1.1428E-04
**AE vs. DAE**	0.0019	4.4563E-56	9.9305E-4	1.3975E-36
**AE vs. DAE-L**	0.0055	5.8103E-253	0.0037	3.5246E-264
**AE vs. SAE-IBS**	0.0057	3.6417E-263	0.0032	1.0222E-224
**AE vs. D-SAE-IBS**	0.0061	6.7913E-281	0.0038	1.0051E-267
**AE vs. D-SAE-IBS-L**	0.0065	1.7604E-301	0.0026	2.3286E-167
**DAE vs. DAE-L**	0.0036	1.6086E-146	0.0027	9.4960E-183
**DAE vs. SAE-IBS**	0.0038	4.7548E-158	0.0022	2.3775E-139
**DAE vs. D-SAE-IBS**	0.0041	2.2379E-178	0.0028	1.0246E-186
**DAE vs. D-SAE-IBS-L**	0.0045	2.4796E-202	0.0016	1.6362E-80
**DAE-L vs. SAE-IBS**	2.0050E-04	1	-4.9524E-04	9.3120E-10
**DAE-L vs. D-SAE-IBS**	5.5732E-04	3.0572E-09	4.6053E-05	1
**DAE-L vs. D-SAE-IBS-L**	9.8614 E-04	3.1105E-16	-0.0012	4.5807E-49
**SAE-IBS vs. D-SAE-IBS**	3.5681 E-04	0.0558	5.4130E-4	1.2833E-11
**SAE-IBS vs. D-SAE-IBS-L**	7.8564 E-04	1.9353E-10	-6.7495E-04	8.5458E-18
**D-SAE-IBS vs. D-SAE-IBS-L**	4.2883 E-04	0.0052	-0.0012	1.6841E-52

Table S14. Results of the two-sample t-test on the NRMSD of different methods over 100 simulations for the experiment of missingness. *Bonferroni correction method was applied to compute the adjusted significance level, accounting for multiple compassion. The non-significant p-values (p>0.0014) are marked in red.*

	Axis1		Axis 2	
	**Mean Difference**	**p-value**	**Mean Difference**	**p-value**
**PCA vs. UPCA**	0.1630	0	0.0639	0
**PCA vs. SUGIBS**	0.1655	0	0.0668	0
**PCA vs. AE**	0.1466	0	0.0504	0
**PCA vs. DAE**	0.1522	0	0.0522	0
**PCA vs. DAE-L**	0.1559	0	0.0627	0
**PCA vs. SAE-IBS**	0.1687	0	0.0705	0
**PCA vs. D-SAE-IBS**	0.1684	0	0.0713	0
**PCA vs. D-SAE-IBS-L**	0.1703	0	0.0718	0
**UPCA vs. SUGIBS**	0.0026	1,9839E-08	0.0029	5,4746E-10
**UPCA vs. AE**	-0.0164	5,7344E-200	-0.0135	6,2688E-146
**UPCA vs. DAE**	-0.0107	1,8379E-111	-0.0117	1,0178E-119
**UPCA vs. DAE-L**	-0.0070	9,9838E-56	-0.0012	0.1654
**UPCA vs. SAE-IBS**	0.0057	1,7269E-38	0.0066	6,1781E-46
**UPCA vs. D-SAE-IBS**	0.0054	8,2382E-35	0.0074	1,2267E-56
**UPCA vs. D-SAE-IBS-L**	0.0073	1,7425E-59	0.0079	4,2976E-64
**SUGIBS vs. AE**	-0.0190	5,2354E-238	-0.0164	1,1732E-189
**SUGIBS vs. DAE**	-0.0133	4,3536E-152	-0.0146	5,9554E-164
**SUGIBS vs. DAE-L**	-0.0096	1,7938E-93	-0.0041	1,4346E-19
**SUGIBS vs. SAE-IBS**	0.0031	1,7951E-12	0.0036	2,0651E-15
**SUGIBS vs. D-SAE-IBS**	0.0028	3,4067E-10	0.0045	8,2071E-23
**SUGIBS vs. D-SAE-IBS-L**	0.0047	2,5271E-27	0.0050	2,2974E-28
**AE vs. DAE**	0.0056	7,0787E-38	0.0017	0.0021
**AE vs. DAE-L**	0.0094	4,5578E-90	0.0122	1,6861E-127
**AE vs. SAE-IBS**	0.0221	1,2341E-281	0.0200	1,2119E-241
**AE vs. D-SAE-IBS**	0.0218	1,6738E-277	0.0208	5,2147E-253
**AE vs. D-SAE-IBS-L**	0.0237	1,2210E-302	0.0214	1,9034E-260
**DAE vs. DAE-L**	0.0037	2,2176E-17	0.0105	1,8995E-101
**DAE vs. SAE-IBS**	0.0164	9,8647E-201	0.0183	1,7633E-217
**DAE vs. D-SAE-IBS**	0.0161	4,5940E-196	0.0191	2,9287E-229
**DAE vs. D-SAE-IBS-L**	0.0180	1,3734E-224	0.0197	5,6104E-237
**DAE-L vs. SAE-IBS**	0.0127	8,7022E-143	0.0078	1,0274E-61
**DAE-L vs. D-SAE-IBS**	0.0124	6,3490E-138	0.0086	2,9361E-73
**DAE-L vs. D-SAE-IBS-L**	0.0143	6,0461E-168	0.0091	3,6164E-81
**SAE-IBS vs. D-SAE-IBS**	-3.0677E-04	1	8.3238E-04	1
**SAE-IBS vs. D-SAE-IBS-L**	0.0016	0.0037	0.0014	0.0430
**D-SAE-IBS vs. D-SAE-IBS-L**	0.0019	1.3777E-04	5.5301E-04	1
